# A virus-induced conformational switch of STAT1-STAT2 dimers boosts antiviral defenses

**DOI:** 10.1038/s41422-020-0386-6

**Published:** 2020-08-05

**Authors:** Yuxin Wang, Qiaoling Song, Wei Huang, Yuxi Lin, Xin Wang, Chenyao Wang, Belinda Willard, Chenyang Zhao, Jing Nan, Elise Holvey-Bates, Zhuoya Wang, Derek Taylor, Jinbo Yang, George R. Stark

**Affiliations:** 1 Department of Cancer Biology, Lerner Research Institute, The Cleveland Clinic Foundation, Cleveland, OH 44195 USA; 2 Marine Drug Screening and Evaluation Platform, Qingdao National Laboratory for Marine Science and Technology, Ocean University of China, Qingdao, Shandong 266071 China; 3 Department of Pharmacology, Case Western Reserve University, Cleveland, OH 44106 USA; 4 Institute of Cancer Biology and Drug Screening, School of Life Sciences, Lanzhou University, Lanzhou, Gansu 730000 China; 5 Key Laboratory of Marine Drugs, Ministry of Education, Ocean University of China, Qingdao, Shandong 266071 China; 6 Department of Immunology, Lerner Research Institute, The Cleveland Clinic Foundation, Cleveland, OH 44195 USA; 7 Proteomics and Metabolomics Laboratory, Lerner Research Institute, The Cleveland Clinic Foundation, Cleveland, OH 44195 USA

**Keywords:** Phosphorylation, Innate immunity, Phosphorylation, Innate immunity

## Abstract

Type I interferons (IFN-I) protect us from viral infections. Signal transducer and activator of transcription 2 (STAT2) is a key component of interferon-stimulated gene factor 3 (ISGF3), which drives gene expression in response to IFN-I. Using electron microscopy, we found that, in naive cells, U-STAT2, lacking the activating tyrosine phosphorylation, forms a heterodimer with U-STAT1 in an inactive, anti-parallel conformation. A novel phosphorylation of STAT2 on T404 promotes IFN-I signaling by disrupting the U-STAT1-U-STAT2 dimer, facilitating the tyrosine phosphorylation of STATs 1 and 2 and enhancing the DNA-binding ability of ISGF3. IKK-ε, activated by virus infection, phosphorylates T404 directly. Mice with a T-A mutation at the corresponding residue (T403) are highly susceptible to virus infections. We conclude that T404 phosphorylation drives a critical conformational switch that, by boosting the response to IFN-I in infected cells, enables a swift and efficient antiviral defense.

## Introduction

Emerging viral threats have recurrently challenged healthcare systems, taking a huge toll on nations and individuals, world-wide. The mammalian innate immune system utilizes the type I interferon (IFN-I) family as the first line of defense against viruses, as well as other types of harmful microbes.^1^ In almost all cell types, IFN-I drives the transcription of IFN-stimulated genes (ISGs),^2^ whose products interfere with different stages of virus infection by many different mechanisms.^3,4^ All subtypes of IFN-I bind to and signal through the IFNAR heterodimeric receptor to activate the receptor-associated tyrosine kinases Janus kinase 1 (JAK1) and tyrosine kinase 2 (TYK2). In turn, these kinases phosphorylate the receptor and then signal transducers and activators of transcription 1 (STAT1) and 2 (STAT2) on specific tyrosine residues, leading to the formation of the main driver of ISG transcription, IFN-stimulated gene factor 3 (ISGF3).

As one of the three components of ISGF3, phosphorylated STAT2 is essential, providing not only the major transactivation domain, but connecting to STAT1 through its Src-homology 2 (SH2) domain, and to IRF9 through its coil-coiled (CC) domain.^5^ In untreated cells, U-STAT1 and U-STAT2 form a very stable, inactive heterodimer, without IRF9, but the structure of this heterodimer is not known.^6,7^ Combining electron microscopic (EM) and biochemical analyses, we demonstrate that U-STAT1 and U-STAT2 assemble in an anti-parallel conformation (U-dimer) that inhibits IFN-I-dependent signaling. The novel phosphorylation of T404 of STAT2 specifically disrupts the U-dimer, facilitating a rapid response to IFN-I, and also greatly enhancing the affinity of ISGF3 for ISG promoters. We further demonstrate that, by activating IKK-ε, viruses elevate T404 phosphorylation. As a consequence, phosphorylation-deficient T403A/T403A mice (corresponding to T404 in humans) have a severely compromised antiviral defense due to an insufficient and delayed transcriptional response to IFN-I. T404 phosphorylation, by driving an inducible conformational switch of the U-STAT1-U-STAT2 heterodimer, enables a much more efficient antiviral response. By using this mechanism, infected cells are more responsive to IFN-I than uninfected cells, promoting a more efficient antiviral response in IFN I-treated infected cells than in IFN I-treated cells that have not yet been infected.

## Results

### Structural analysis of U-STAT1-U-STAT2 heterodimers (U-dimer) by EM

**Fig. 1:**
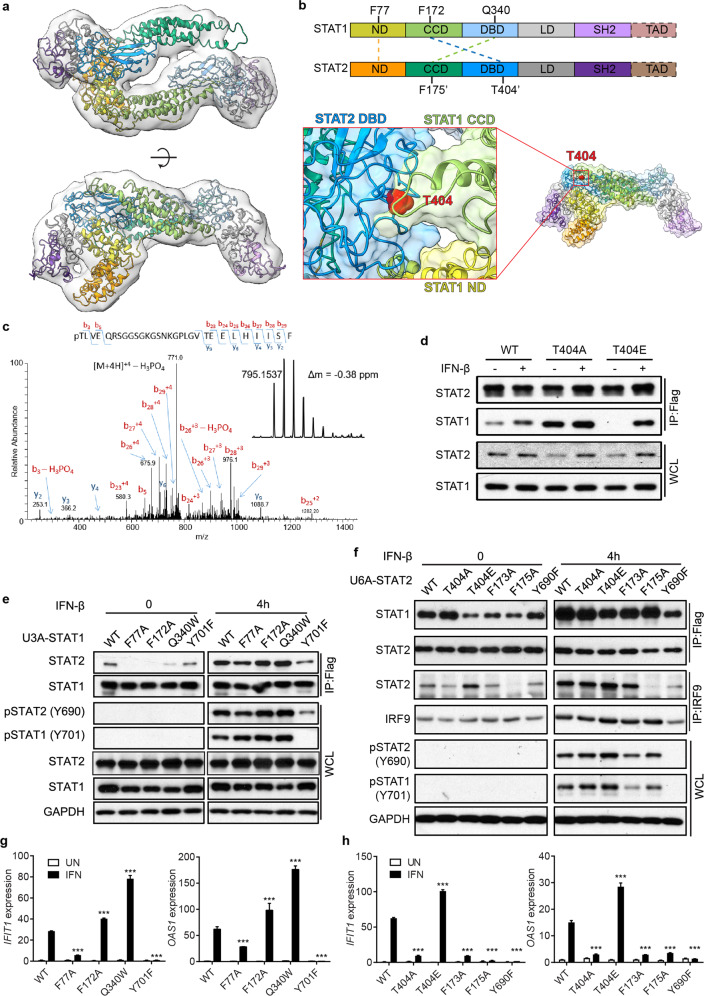
Phosphorylation of STAT2 on T404 disrupts the U-STAT1-U-STAT2 dimer, a negative regulator of IFN-I-dependent signaling. **a** EM density at contour level of 1.81 shown in transparent surface. Proteins are depicted as cartoon models. **b** Schematic diagram showing the key residues for STAT1-STAT2 dimer, and close-up view of T404 in STAT1-STAT2 interface. **c** MS/MS spectra for the phosphorylated chymotryptic peptide pTLVEQRSGGSGKGSNKGPLGVTEELHIISF. This quadropoly charged peptide has an observed m/z of 795.1537 Da and is within −0.38 ppm of the expected mass. This spectrum is dominated by H_3_PO_4_ loss, consistent with the presence of a phosphorylated S or T residue. The masses of the b_3_ and b_5_ ions are consistent with phosphorylation at T404. **d** STAT2-null U6A cells expressing WT, T404A, or T404E STAT2 were treated with IFN-β (100 IU/mL) for 30 min or untreated. Whole-cell lysates were used for immunoprecipitations of Flag-STAT2. **e** STAT1-null U3A cells expressing WT, F77A, F172A, Q340W, or Y701F STAT1 were treated with IFN-β (100 IU/mL) for 4 h or untreated. Whole-cell lysates were used for immunoprecipitations of Flag-STAT1, and analyzed by western blot. **f** U3A cells expressing WT, F77A, F172A, Q340W, or Y701F STAT1 were treated with IFN-β (100 IU/mL) for 4 h or untreated. Total RNAs were analyzed by qRT-PCR. **g** U6A cells expressing WT, T404A, T404E, F173A, F175A, or Y690F STAT2 were treated with IFN-β (100 IU/mL) for 4 h. Whole-cell lysates were used for immunoprecipitations of Flag-STAT2 or IRF9 and analyzed by western blot. **h** U6A cells expressing WT, T404A, T404E, F173A, F175A, or Y690F STAT2 were treated with IFN-β (100 IU/mL) for 4 h or untreated. Total RNAs were analyzed by qRT-PCR. Data are shown as means ± SEM from three independent experiments. *P* values were calculated using the paired ratio *t*-test (mutants vs WT, ***P < 0.001).

We purified U-STAT2 and U-STAT1 from mammalian cells stably expressing the FLAG-tagged proteins and imaged the chromatographic fraction corresponding to the heterodimer by electron microscopy (EM), following negative staining (Supplementary information, Fig. S1a). The EM map unambiguously demonstrates that the U-dimer has an anti-parallel conformation (Fig. 1a), and reveals that its conformation resembles the previously determined structure of the anti-parallel U-STAT1 homodimer.^8,9^ Based on the known structure of the U-STAT1 homodimer (PDB entry: 1YVL) and the three-dimensional reconstructed EM map,^9^ we modeled the conformation of the U-dimer (Fig. 1a). Our current EM map, despite in low resolution, enables us to observe three protein-protein interfaces that stabilize the anti-parallel conformation, including STAT1-ND (N-terminal domain)-STAT2-ND, STAT1-CCD (coiled-coil domain)-STAT2-DBD (DNA-binding domain), and STAT1-DBD-STAT2-CCD (Fig. 1b). To validate this structural model, we designed several truncated versions of STAT1 and STAT2 and used glutathione S-transferase (GST) pull-down assays to examine the formation of heterodimer, confirming that STAT2 interacts mainly through the DBD of STAT1, and vice versa (Supplementary information, Fig. S1b, c). Since the anti-parallel conformation represents an inhibitory state of this STAT complex, the question of how this inhibitory state is disfavored arises. Interestingly, a post-translational modification (PTM) site, T404, identified in our mass spectrometry analysis, resides in the core of the heterodimeric interfaces (Fig. 1b, c). We expect that the addition of a bulky, negatively-charged phosphate group would disrupt the stable STAT1-STAT2 heterodimer interfaces, providing a clear example of how PTMs might be critical regulators of IFN-I-dependent signaling.

### Phosphorylation of STAT2 T404 disfavors the inactive U-dimer

To investigate the effect of T404 phosphorylation on the formation of the inactive U-dimer, we mutated this residue to alanine, preventing its phosphorylation, and to glutamate, mimicking the negative charge of phospho-threonine. Without IFN-I treatment, the binding of U-STAT1 to U-STAT2 was greatly enhanced by the T404A mutation of STAT2 (Fig. 1d) and severely inhibited by the T404E mutation. In IFN-treated cells, there was no effect of these mutations on the stability of the complex between tyrosine-phosphorylated STAT1 and STAT2. EM analysis revealed that the U-dimer with a T404A mutation of STAT2 has the same conformation as the U-dimer with WT (wild-type) STAT2 (Supplementary information, Fig. S1d), confirming that preventing the phosphorylation of T404 stabilizes the anti-parallel conformation of the U-dimer.

To further investigate how U-STAT1 and U-STAT2 interact in this anti-parallel conformation, we studied the effects of a series of mutations of STAT1 on U-dimer stability. F77A (an ND mutation), F172A (a CCD mutation), and Q340W (a DBD mutation) abolished U-dimer formation, while Y701F (a tyrosine phosphorylation-deficient mutation) had no effect (Fig. 1e). We also found that the expression of canonical ISGs (*IFIT1* and *OAS1*) was enhanced by the F172A and Q340W mutations, which destabilize the anti-parallel conformation (Fig. 1f). Notably, because the tyrosine phosphorylation of STAT1 and STAT2 was delayed by the F77A mutation of STAT1 (Supplementary information, Fig. S1e), ISG induction was compromised upon IFN-I treatment. We conclude that, although the ND-domain and CCD-DBD interactions are both critical for stabilizing the U-dimer, only the mutations that affect the CCD-DBD interactions specifically disturb the anti-parallel, inactive U-dimer and thus enhance IFN-I responses.

We propose that U-STAT1 associates with U-STAT2 through F173 and F175 in the CCD and T404 in the DBD of U-STAT2. The F175A mutant of U-STAT2 fails to bind to U-STAT1 and IRF9, suggesting that U-STAT1 and IRF9 compete for binding to U-STAT2 through this residue.^5^ The T404A mutant of U-STAT2 has a stronger interaction with U-STAT1, but a weaker interaction with IRF9, while the effect of the T404E mutation of U-STAT2 on its interactions with STAT1 and IRF9 is completely opposite (Fig. 1g). In our model of the U-dimer, F173 of STAT2 is located in the CC domain near F175, but facing inward, suggesting that the inhibition of ISGF3 function seen with the F173A mutant might be due to a local conformational change. Consistent with this idea, the response to IFN-β is severely compromised in U6A cells expressing the mutants T404A or F175A (Fig. 1h). These results reveal that F175 of U-STAT2 is important for its interaction with both U-STAT1 and IRF9, and that T404 phosphorylation is the switch for a conformational change of U-dimer, which is a potent negative regulator of IFN-I-dependent signaling.

### Phosphorylation of STAT2 on T404 enhances the affinity of ISGF3 for DNA

**Fig. 2:**
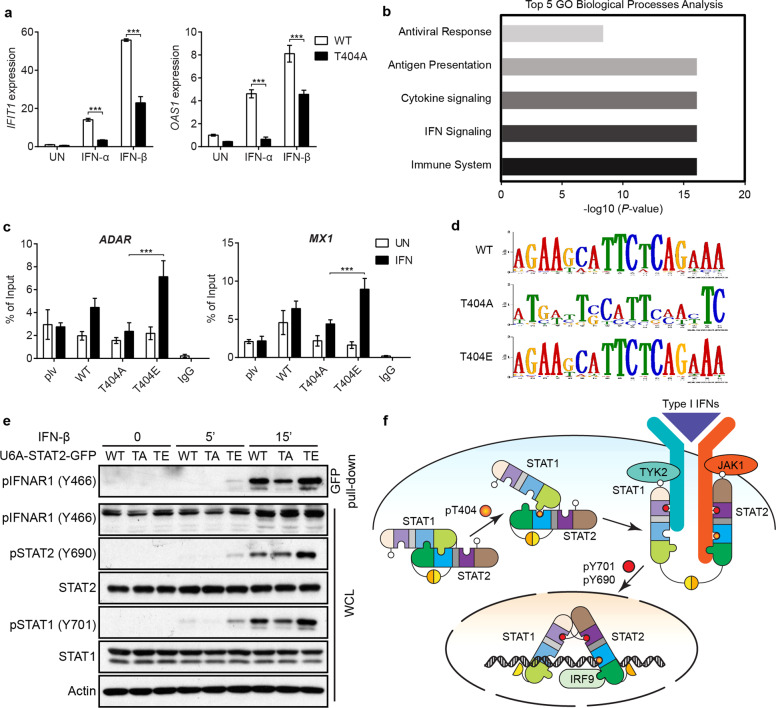
Phosphorylation of STAT2 on T404 expedites the tyrosine phosphorylation of STAT1 and STAT2, and enhances the DNA-binding activity of ISGF3. **a** U6A cells expressing WT or T404A STAT2 were treated with IFN-α or IFN-β (100 IU/ml). Cells were harvested after 4 h and total RNAs were analyzed by qRT-PCR. **b** U6A cells expressing WT or T404A STAT2 were treated with IFN-β for 0, 4, 8, or 24 h. Total RNAs were analyzed by using an Illumina HumanHT-12 v4 Expression BeadChip array. The average signal for each probe was used to determine expression levels. Genes with detection *P* values greater than 0.01 in the untreated or treated cells were excluded from the analysis. Inductions of less than 2-fold were not scored. A gene ontology (GO) analysis was performed of genes differentially expressed between U6A cells expressing T404A or WT STAT2, stimulated with IFN-β for 4, 8, or 24 h. **c** The occupancy of the *ADAR1* and *MX1* promoters by IRF9 in U6A cells expressing WT, T404A, or T404E STAT2, treated with IFN-β (100 IU/mL), was assayed by ChIP, using anti-IRF9. **d** Samples from **c** were analyzed by ChIP-seq. The top 100 segments of each sample were used as inputs. Motifs were discovered by MEME v5.0.3. **e** U6A cells expressing GFP-tagged WT, T404A, or T404E STAT2 were treated with IFN-β (100 IU/mL) for 0, 5, or 15 min. Whole-cell lysates were used for GFP pull-down and were analyzed by western blot. **f** Working model shows that, T404 phosphorylation, by destabilizing the U-STAT1-U-STAT2 anti-parallel dimer, increases the affinity of STAT2 for activated IFNAR1, expedites the tyrosine phosphorylation of both STAT2 and STAT1, and enhances the affinity of ISGF3 for ISREs. Data are shown as means ± SEM from three independent experiments. *P* values were calculated using the paired ratio *t*-test (***P < 0.001).

To further investigate the functional outcomes of T404 phosphorylation, we analyzed the expression of some typical ISGs in response to IFN-I in STAT2-null U6A cells in which STAT2 variants are expressed. The T404A mutation dramatically inhibited the induction of *IFIT1* and *OAS1* after treatment with either IFN-α or IFN-β (Fig. 2a). We also analyzed the kinetics of IFN-β-induced gene expression, finding that the induction of these ISGs was greatly inhibited in cells expressing T404A at all times, especially after 8 h (Supplementary information, Fig. S2a). Compared to the WT protein, T404E STAT2 mediated more ISG induction in response to IFN-β, contrasting with the effect of the T404A mutation, while T404D partially mimicked the effect of T404 phosphorylation (Supplementary information, Fig. S2b).

We used an Illumina Gene Expression Array to understand in more detail how T404 phosphorylation affects ISG induction. Of the 31,425 genes on the array, 181 were induced in cells containing WT STAT2 after 4 h, 210 after 8 h, and 227 after 24 h. Of the total of 358 genes that responded to treatment with IFN-β at all three time periods, 266 were induced less well in cells with the T404A mutant than in cells with WT STAT2. A gene ontology analysis revealed that genes induced less well in U6A-T404A-STAT2 cells fell into sub-groups whose products are involved in antiviral defense, immune responses, or the IFN-I signaling pathway (Fig. 2b). Of these genes, 55 (Supplementary information, Fig. S2c) were affected at all three time periods. Most of them are well-known ISGs (Supplementary information, Table S1), with canonical interferon-stimulated response elements (ISREs) in their promoters (Supplementary information, Fig. S2d). Since the expression of most ISGs is inhibited by the T404A mutation, we conclude that the phosphorylation of STAT2 on T404 has a very substantial positive effect on the transcriptional activity of ISGF3.

We investigated whether the phosphorylation of STAT2 on T404 stabilizes ISGF3 and enhances its ability to bind to DNA. IRF9 is primarily responsible for the binding of ISGF3 to ISREs. In a chromatin immunoprecipitation (ChIP) assay with anti-IRF9, we found that, following treatment with IFN-β, the recruitment of IRF9 to ISREs was not induced in control U6A cells, which lack STAT2 expression, as expected (Fig. 2c). IRF9 in company with WT STAT2 was well recruited to ISREs, but was recruited much more strongly with ISGF3 variants containing T404E, but not T404A STAT2. We performed a ChIP-seq analysis with the above samples, followed by next-generation sequencing. Shown in Fig. 2c are the top ranked binding motifs for each sample, determined by using the Multiple Em for Motif Elicitation (MEME) algorithm.^10^ We observed binding to a 5′-TTCTCAGAAA-3′ motif in IFN-β-treated cells expressing WT or T404E STAT2, but not T404A STAT2 (Fig. 2d). We also used electrophoretic mobility shift assays (EMSAs) with an ISRE probe to assess the ability of ISGF3 to bind to DNA. In U6A cells that express WT STAT2, ISGF3 was detected readily. The amount of ISGF3 capable of binding to an ISRE sequence is greatly enhanced by the T404E mutation of STAT2 and severely inhibited by the T404A mutation (Supplementary information, Fig. S2e). We conclude that the phosphorylation of T404, which lies in the DNA binding domain of STAT2, enhances the affinity of ISGF3 for ISREs. Previously, we found that the phosphorylation of STAT2 T387 has an effect opposite to that of the phosphorylation of T404 in IFN-I-dependent signaling, and in U-STAT1-STAT2 formation.^6^ However, the T-to-D mutation of T387 does not mimic the phosphorylation, indicating that the effect of T387 phosphorylation is not mediated by the negative charge, and suggesting that T387 phosphorylation functions through a specific mechanism, e.g., by recruiting one or more proteins that regulate the balance between U-STAT1-STAT2 and ISGF3.

### Phosphorylation of STAT2 on T404 expedites the tyrosine phosphorylation of STAT1 and STAT2

We investigated whether the phosphorylation of STAT1 and STAT2 on specific tyrosine residues, required for IFN-I-dependent signaling, is affected by the status of the U-dimer, which is destabilized by the phosphorylation of T404. Phosphorylation of T404 enhanced the tyrosine phosphorylation of STAT1 and STAT2 at the early time of 30 min, and the level of tyrosine phosphorylation reached a plateau after 6 h (Supplementary information, Fig. S2f). A similar result was observed with HME (human mammary epithelial) cells expressing WT, T404A, or T404E STAT2 (Supplementary information, Fig. S2g). Since it has been shown that the anti-parallel U-STAT1 homodimer contributes to the de-phosphorylation of Y701 of STAT1,^8^ we investigated whether the differences in tyrosine phosphorylation of STAT1 and STAT2 might be due to effects on de-phosphorylation. Cells were treated with IFN-β for 2 h, followed by treatment with Staurosporine, a kinase inhibitor that rapidly blocks tyrosine phosphorylation. This procedure permits analysis of the rate of decay of the initially phosphorylated residues. The T404E mutation did not suppress the de-phosphorylation of tyrosine residues of either STAT1 or STAT2 (Supplementary information, Fig. S2h). In addition, an assay for nuclear translocation of the STATs showed that T404 phosphorylation did not affect this process either (Supplementary information, Fig. S2i). Taken together, our results reveal that the phosphorylation of STAT2 on T404 enhances the tyrosine phosphorylation of STAT1 in response to IFN-I, especially at early time.

The IFN-I receptor consists of two subunits, IFNAR1 and IFNAR2. Following ligand binding, IFNAR1 is phosphorylated on tyrosine 466, which recruits STAT2 (but not STAT1) via its SH2 domain,^11^ followed by the sequential phosphorylation, first of STAT2 and then of STAT1.^12^ We investigated how STAT2 T404 phosphorylation affects the tyrosine phosphorylation of STAT1 and STAT2, observing that the T404A mutation of STAT2 reduces and delays the interaction of STAT2 with phosphorylated IFNAR1, whereas the T404E mutation leads to a stronger interaction following treatment with IFN-I (Fig. 2e). These results indicate that T404 phosphorylation enhances and expedites the tyrosine phosphorylation of both STAT2 and STAT1, increasing the affinity of STAT2 for activated IFNAR1 by destabilizing the U-STAT1-U-STAT2 dimer (Fig. 2f).

### Virus infection induces the T404 phosphorylation of STAT2 by IKK-ε

**Fig. 3:**
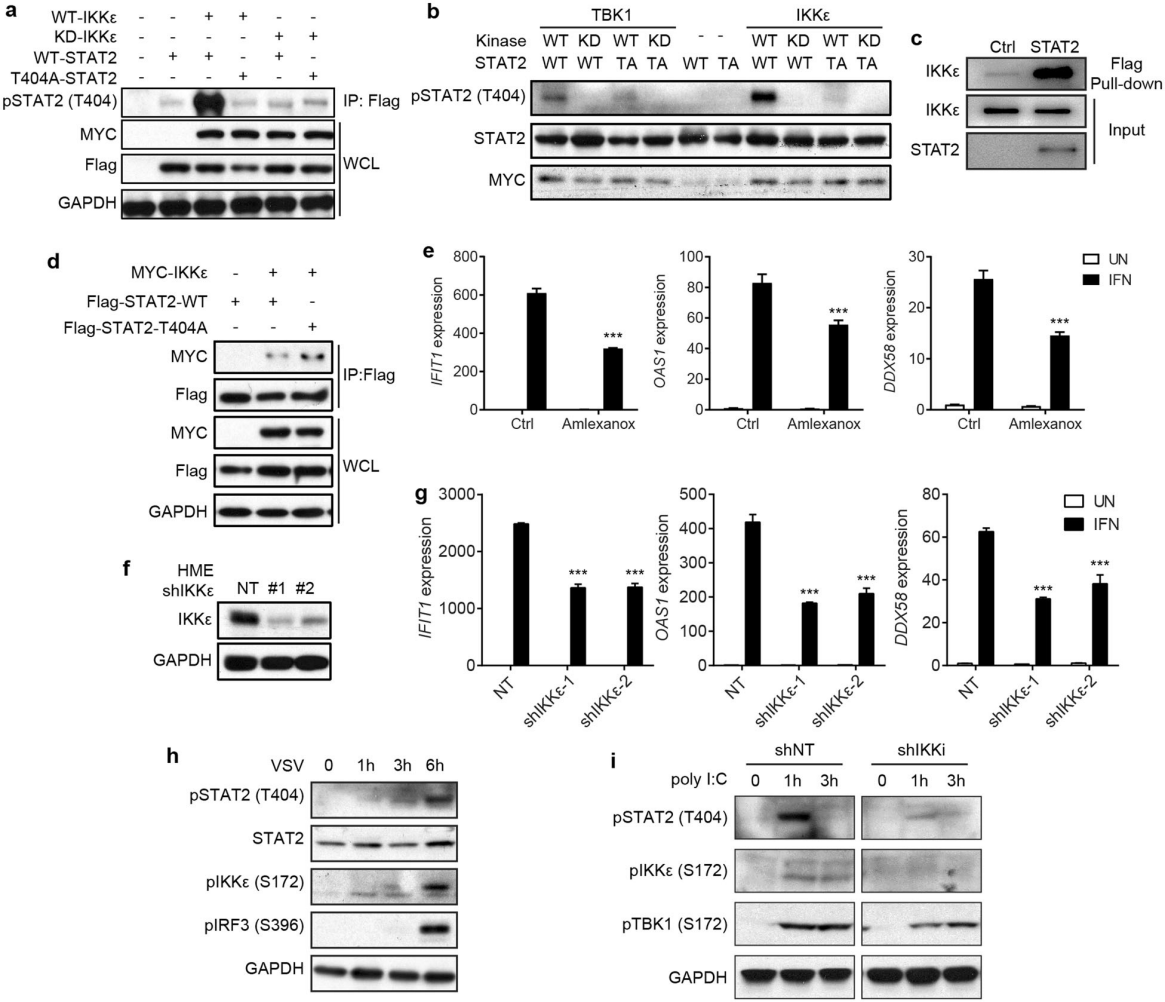
Virus infection induces T404 phosphorylation of STAT2 by IKK-ε. **a** HEK293T cells were co-transfected with IKK-ε and STAT2 constructs as indicated. Whole-cell lysates were used for immunoprecipitations of Flag-STAT2, and analyzed by western blot. **b** The indicated samples from an in vitro kinase assay were analyzed by western blot. Kinases were tagged with MYC, and STAT2 were tagged with Flag as substrate. **c** Purified Flag-STAT2 was mixed with commercial IKK-ε, immunoprecipitated with anti-Flag, then immunoblotted with anti-IKK-ε. **d** HEK293T cells were co-transfected with IKK-ε and STAT2 as indicated. Whole-cell lysates were used for immunoprecipitations of Flag-STAT2, and analyzed by western blot. **e** HME cells were pretreated with amlexanox (2 μM) for 30 min, followed by IFN-β (100 IU/mL) for 4 h. Total RNA was analyzed by qRT-PCR. **f** Whole-cell lysates from HME cells with expression of shRNAs targeting IKK-ε were analyzed by western blot. **g** HME cells with expression of shRNAs targeting IKK-ε were treated with IFN-β (100 IU/mL) for 4 h. Total RNA was analyzed by qRT-PCR. **h** BJ (primary human foreskin fibroblasts) cells were infected with VSV (MOI = 1) for 1, 3 or 6 h. Whole-cell lysates were analyzed by western blot. **i** Hela cells were transfected with poly I:C (10 ng/mL) for 1 h, or 3 h. Whole-cell lysates were analyzed by western blot. Data are shown as means ± SEM from three independent experiments. *P* values were calculated using the paired ratio *t*-test (***P < 0.001).

We generated a specific polyclonal antibody against a STAT2 peptide that includes phosphorylated T404 and used ELISA to demonstrate that this antibody does not cross-react with the corresponding un-phosphorylated peptide (Supplementary information, Fig. S3a). To identify kinases responsible for STAT2 T404 phosphorylation, we found that the amino acid sequence surrounding T404 in STAT2 is a good match with sequences that are preferentially phosphorylated by IKK-ε. Phosphorylation of STAT1 on S708 by IKK-ε is known to regulate the formation of STAT1 homodimers in response to IFN-γ, but not ISGF3 formation in response to IFN-I.^13,14^ Western analyses employing this antibody showed that the phosphorylation of STAT2 on T404 is induced by ectopic expression of IKK-ε, but not a kinase-dead mutant enzyme (Fig. 3a). We also used an in vitro kinase assay to demonstrate that IKK-ε phosphorylates T404 of STAT2 directly (Fig. 3b). Confirming this result, the phosphorylation of STAT2 was greatly impaired when T404 was mutated. We also evaluated TBK1, a homolog of IKK-ε, finding that it can also phosphorylate STAT2 on T404, although not as robustly as IKK-ε (Fig. 3b). To obtain more information on how these kinases phosphorylate STAT2 on T404, we performed in vitro pull-down assays with purified IKK-ε and TBK1. STAT2 binds directly to both IKK-ε (Fig. 3c) and TBK1 (Supplementary information, Fig. S3b). Using a set of GST-fusion proteins with different IKK-ε truncations, we detected a direct interaction between the kinase domain of IKK-ε and STAT2 (Supplementary information, Fig. S3c), and also found that the STAT2 DBD, which includes T404, binds directly to IKK-ε (Supplementary information, Fig. S3d). In addition, a co-immunoprecipitation experiment using HEK293T cells with ectopic expression of both STAT2 and IKK-ε revealed that the T404A mutation did not abrogate their interaction (Fig. 3d). These data indicate that IKK-ε binds directly to the DBD of STAT2 to phosphorylate T404 as an important component of a robust response to IFN-I, while TBK1 has a weaker ability to phosphorylate this residue.

Pretreatment of HME cells with Amlexanox, an IKK-ε inhibitor, dramatically inhibited the induction of ISGs by IFN-I (Fig. 3e). Furthermore, knocking down the expression of IKK-ε in the same cells with an shRNA (Fig. 3f) significantly suppressed ISG expression in response to IFN-β (Fig. 3g). We observed similar results when the same experiment was done with Hela cells (Supplementary information, Fig. S3e, f). We also noted that down-regulation of TBK1 expression, driven by an shRNA, also suppressed ISG expression (Supplementary information, Fig. S3g, h).

IKK-ε and TBK1 phosphorylate and activate IFN regulatory factor 3 (IRF3) in response to virus infection, further evoking cellular antiviral responses, including the production of IFN-I.^15–17^ We found that, in human primary fibroblast cells, VSV strongly induced the phosphorylation of STAT2 on T404, along with that of IRF3 (Fig. 3h), and the same observation was made in HME cells (Supplementary information, Fig. S3i). Poly I:C, which simulates virus infections, significantly induced T404 phosphorylation, and this activity was abolished by silencing IKK-ε (Fig. 3i). We conclude that virus infections induce T404 phosphorylation in an IKK-ε-dependent manner.

### The phosphorylation of STAT2 on T404 is critical for IFN-dependent antivirus defenses in vitro and in vivo

The amino acid sequence surrounding T404 in human STAT2 is highly conserved in several other mammalian species (Supplementary information, Fig. S4a). We generated STAT2 T403A/T403A mice, using CRISPR/Cas9 technology (Supplementary information, Fig. S4b, c), in order to examine the effects of the T404 mutation on virus defense in vivo (the corresponding murine residue is T403). Signaling in response to IFN-I was assessed in murine embryonic fibroblasts (MEFs) and in bone marrow-derived dendritic cells (BMDCs) from STAT2 WT/WT or T403A/T403A mice. Four hours after treatment with murine IFN-β, the induction of ISGs was greatly reduced in cells derived from T403A/T403A mice (Supplementary information, Fig. S4d, e).

**Fig. 4:**
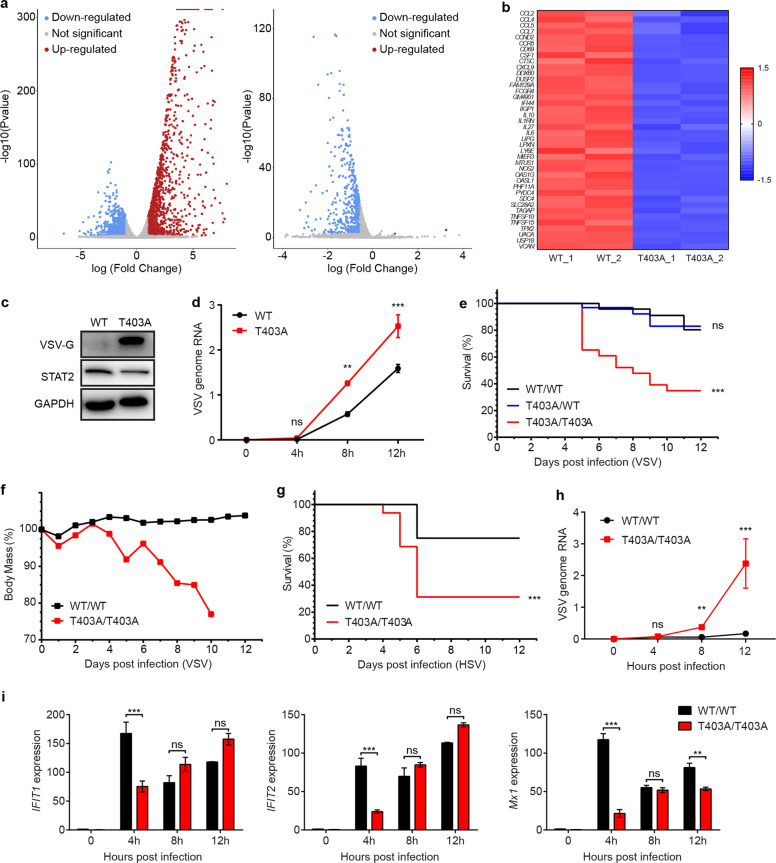
Phosphorylation of STAT2 on T404 is critical for IFN-dependent antiviral defense. **a** Volcano plots for differential gene expression following IFN-β treatment (6 h) of bone marrow-derived macrophages (BMDMs). WT/WT treated vs untreated (left), T403A/T403A treated vs WT/WT treated (right). Transcripts with fold changes > 2 and *P* < 0.01 after FDR adjustment were defined as differentially expressed genes (DEGs) and are highlighted with blue for down-regulated and red for up-regulated DEGs. **b** Heatmap of the top 40 DEGs in IFN-β-treated (6 h) BMDMs derived from WT/WT or T403A/T403A mice. **c** Primary MEFs with WT or T403A STAT2 were treated with 10 IU/mL IFN-β for 2 h, then infected with VSV-GFP (MOI = 1) for 2 h, the expression of VSV G protein was analyzed by the western blot at 12 h post infection. **d** Primary MEFs with WT or T403 STAT2 were infected with WT VSV (MOI = 1) for 2, 4, 8, or 12 h, and the amounts of VSV genomic RNA (VSV-N) were analyzed by qRT-PCR. **e** Eight-week-old WT/WT, STAT2 T403A/WT, or STAT2 T403A/T403A mice were infected intravenously with 1 × 10^7^ pfu of VSV for 12 days, and their survival was recorded (*n* = 20). *P* < 0.0001 for the difference between WT/WT and T403A/T403A. **f** Mice from the experiment in **c** were weighed daily (*n* = 20 mice per cohort per dose). Values represent average scores of overall weight loss compared with initial body mass. **g** Eight-week-old WT/WT, T403A/WT, or T403A/T403A mice were infected intravenously with 8 × 10^5^ pfu of HSV for 12 days, and their survival was recorded (*n* = 16). *P* < 0.0001 for the difference between WT/WT and T403A/T403A. **h** WT/WT and T403A/T403A mice (*n* = 4) were infected with VSV for 4, 8, or 12 h and the amount of VSV genomic RNA in the spleens was analyzed by qRT-PCR. **i** ISG induction in the spleens from **f** was analyzed by qRT-PCR. Data are shown as means ± SEM from two independent experiments. *P* values were calculated using the paired ratio *t*-test (**P < 0.01; ***P < 0.001, ns, not significant).

The expression of about 74% (266/358) of ISGs induced by IFN-I was reduced in U6A cells expressing exogenous T404A STAT2 (Supplementary information, Fig. S2c). To determine how T404 phosphorylation affects IFN-I signaling in cells expressing endogenous STAT2, especially immune cells, we used RNA-seq to performed transcriptome profiling in macrophages derived from WT/WT or T403A/T403A mice. Most of the 606 genes that are well-induced (fold change > 2) in WT cells after stimulation with IFN-I (Fig. 4a, left) are induced much less well in T403A cells (Fig. 4a, right). The top 40 genes are listed in Fig. 4b. A gene ontology enrichment analysis demonstrated that the genes with lower expression in T403A cells can be categorized into cytokine activity, adaptive immune response, and myeloid leukocyte activation (Supplementary information, Fig. S4f).

The protein products of IFN-induced ISGs are critical for host cell defense against virus infection. To determine whether the differences in gene expression between WT and T404A STAT2 cells translate into a compromised antiviral defense, MEFs from WT/WT or T403A/T403A mice were infected with VSV-GFP. The amount of VSV-G protein in T403A/T403A MEFs was substantially higher than in WT/WT MEFs (Fig. 4c; Supplementary information, Fig. S5a). We also monitored the kinetics of infection with WT VSV (Fig. 4d). The difference in the amount of VSV genomic RNA between MEFs derived from WT/WT and T403A/T403A mice became more dramatic with time. We repeated the same experiment in HME cells, in which the expression of STAT2 is constitutively low, stably expressing ectopic FLAG-tagged WT, T404A, or T404E STAT2 at comparable levels.^6^ The T404E cells were more resistant to VSV infection, while cells expressing T404A STAT2 were more susceptible than cells expressing WT STAT2 (Supplementary information, Fig. S5b). These results indicate that the phosphorylation of STAT2 on T404 is required for full antiviral activity in both human and murine cells.

To determine whether the phosphorylation of STAT2 on T403 is crucial for protection against infection in vivo, WT/WT, WT/T403A, and T403A/T403A mice were challenged with VSV, administered by tail vein injection. The T403A/T403A mice were highly susceptible to infection, while their WT siblings were resistant (Fig. 4e). T403A/WT mice, in which both forms of STAT2 are expressed, responded similarly to WT/WT mice, suggesting that STAT2 phosphorylated on T403 is vital in the defense against this infection. T403A/T403A mice are also much more sensitive to exposure of VSV in terms of weight change following infection (Fig. 4f). The body masses of T403A/T403A mice started to drop on day 4 post infection and continued thereafter, while the WT/WT mice started to recover at this time. We also observed that T403A/T403A mice developed conjunctivitis (Supplementary information, Fig. S5c). In addition to VSV, an RNA virus, we also challenged the mice with HSV, a DNA virus, and found that T403 phosphorylation of STAT2 is essential for antiviral defense in vivo (Fig. 4g).

### Impaired virus clearance, due to a compromised IFN response, leads to severe viral encephalitis in T403A/T403A mice

Viral infection activates host immune responses and induces the expression of IFNs and other cytokines. When the mice were challenged with VSV for a short time, the amount of IFN-β induced was higher in most of the tissues from T403A/T403A mice (Supplementary information, Fig. S5d), showing that the susceptibility of these mice to VSV is not due to a deficiency in the production of IFN-I. We also determined how infection correlated with induction of the IFN response, by checking the expression of ISGs in spleens, as the lymphoid organ most critical for detection and response to infection. The level of VSV genomic RNA was dramatically higher in the tissues of T403A/T403A mice than in WT/WT mice (Fig. 4h). Interestingly, the expression of ISGs induced by infection was much lower in the former, especially at early time points (Fig. 4i). The above data is consistent with our previous finding (Fig. 2f), indicating that T404/403 phosphorylation expedites antiviral defense by enhancing responses to IFN-I.

**Fig. 5:**
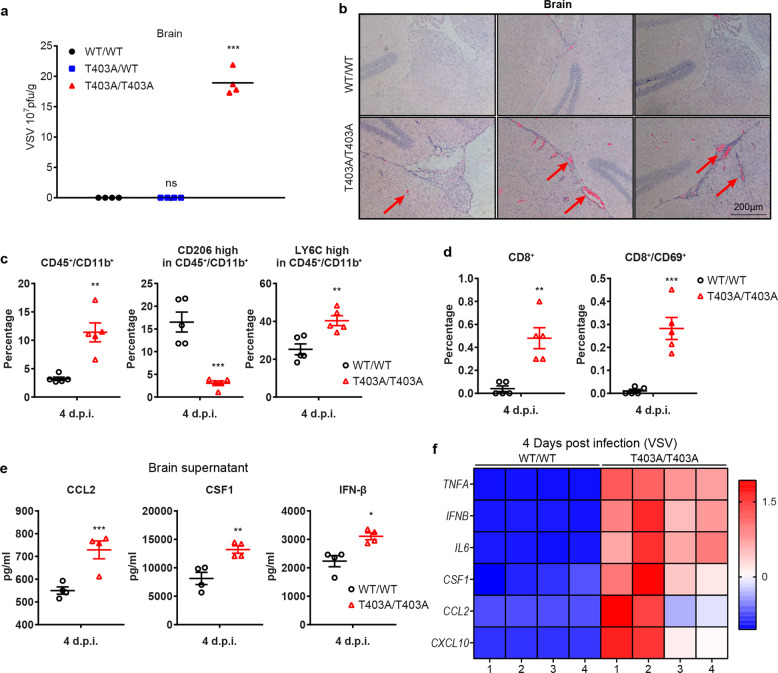
Impaired virus clearance leads to severe viral encephalitis in T403A/T403A mice. **a** WT/WT, T403A/WT, and T403A/T403A mice (*n* = 4) were infected with VSV for 5 days and VSV titers were measured. **b** Differential pathology of WT/WT and T403A/T403A mice in response to VSV. Shown are brain sections stained with hematoxylin and eosin from the mice described in **a**. Scale bar, 200 μm. **c**, **d** WT or STAT2 T403A mice were injected intravenously with 1 × 10^7^ pfu of VSV or buffer. Four days post infection, the brains were excised and analyzed by flow cytometry. **e** Plasma from **c** were analyzed by ELISA. **f** Brains from **c** were analyzed by qRT-PCR. Data are shown as means ± SEM from two independent experiments. *P* values were calculated using the paired ratio *t*-test (*P < 0.05; **P < 0.01; ***P < 0.001; ns, not significant).

Although VSV infects a variety of tissues, neurons are a major target. We monitored the levels of viral genomic RNA in different tissues six days after infection (Supplementary information, Fig. S5e). Very little viral RNA was detected in the brains of WT/WT mice, which had already recovered from the infection at this time (Supplementary information, Fig. S5e). Titers of VSV were about 1000-fold higher in the brains of the T403A/T403A mice (Fig. 5a). To further characterize the consequences of VSV infection, we stained different tissues with hematoxylin and eosin, revealing significant differences in the brains (Fig. 5b), but not in other tissues (Supplementary information, Fig. S5f). A dramatic lesion in the brains of the T403A/T403A mice was perivascular cuff, defined as an accumulation of lymphocytes or plasma cells in a dense mass around blood vessels. Cuffing is usually seen in the margins of plaques, where it serves as a marker of inflammation.^18^ We used flow cytometry to quantify the numbers of immune cells, as indications of inflammation,^19^ in the brains four days after infection. The percentages of CD45^hi^CD11b^hi^ cells were substantially higher in infected T403A/T403A mice (Fig. 5c, left). Within this infected population, the percentages of cells expressing high levels of CD206, an anti-inflammatory marker, were much lower (Fig. 5c, middle), and the percentages of cells with high expression of Ly6C, a pro-inflammatory marker, were much higher in T403A/T403A than WT/WT mice (Fig. 5c, right). These results reveal that many myeloid cells had infiltrated into the brains of T403A/T403A mice, promoting local inflammation at a late stage of VSV infection, due to failure to remove the virus. In encephalitis, T cells are viewed primarily as detrimental.^20^ CD8^+^ cytotoxic T cells play an especially important role in viral encephalitis, helping to eliminate the virus along with infected cells. CD8^+^ T cells derived from T403A/T403A mice had much higher expression of CD69, a T cell activation marker, than T cells derived from WT/WT mice (Fig. 5d), while CD4^+^ T cells and CD19^+^ B cells remained similar (Supplementary information, Fig. S5g).

Along with massive inflammatory cell infiltration, elevated proinflammatory cytokine and chemokine expression, called a cytokine storm, result in deleterious encephalitis that is caused by rapid virus replication.^18,21^ We found significantly higher levels of CCL2 and CSF1, myeloid cell-derived chemokines, in the brain supernatants and plasma from T403A/T403A mice, along with robust production of IFN-β (Fig. 5e and Supplementary information, Fig. S5h). We also observed excessive expression of classical cytokine storm-associated cytokines and chemokines in the brain tissues (Fig. 5f). Together with our previous observation of a delayed IFN response (Fig. 4i), the mice with T403 phosphorylation deficiency failed to release U-STAT1 and U-STAT2 from the inactive anti-parallel conformation to facilitate a rapid antiviral response, which makes them vulnerable to viral encephalitis with an overactive immune response and an intractable cytokine storm.

## Discussion

**Fig. 6:**
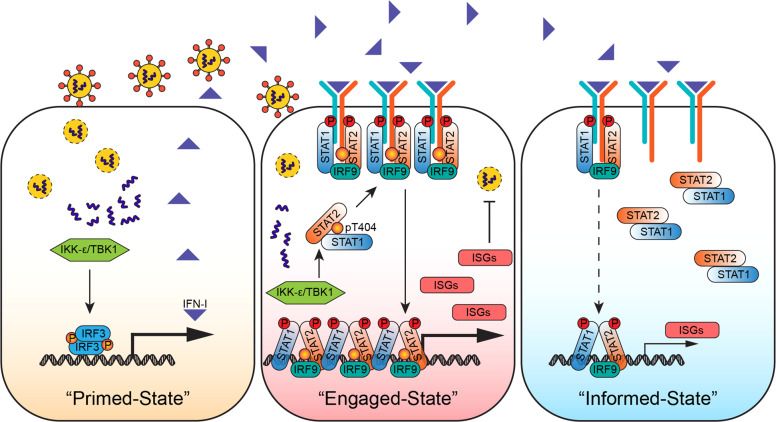
A virus-induced conformational switch of STAT1-STAT2 dimers, driven by T404 phosphorylation of STAT2, boosts IFN-mediated antiviral defenses. To initiate a systematic antiviral immune response, virus-infected cells produce IFN-I (Left, “Primed-State”), along with induction of STAT2 T404 phosphorylation, which engages an enhanced IFN response to eradicate viruses and the infected cells more efficiently (Middle, “Engaged-State”). Unlike infected cells, distant uninfected cells will be informed by IFN-I to be on guard against potential virus infection (Right, “Informed-State”), but without excessive response.

Our model shows that virus infection, along with the production of IFN-I (Fig. 6, left), induces T404 phosphorylation, which primes infected cells to engage an antiviral state, boosting IFN responses to aid on removing the infected cells more efficiently (Fig. 6, middle). Distant uninfected cells will be informed by IFN-I to be on guard against potential virus infection (Fig. 6, right), but without engaging the most extreme response that is needed for infected cells. In this scenario, T404 phosphorylation engages a conformational switch of U-STAT1-U-STAT2 dimers, to finely coordinate IFN-mediated antiviral defenses, not only by enabling efficient eradication of viruses, but also by preventing an overreaction to IFN-I in uninfected cells.

STAT2 is a key component of ISGF3 and also enables the tyrosine phosphorylation of STAT1 by the IFN-I receptor.^22^ However, if the amount of U-STAT2 exceeds that of U-STAT1, the very stable U-STAT1-U-STAT2 dimers will inhibit the response of cells to STAT1-dependent cytokines, including IFN-γ.^7^ Similarly, if the amount of U-STAT1 exceeds that of U-STAT2, the amount of the transcriptionally active U-STAT2-IRF9 dimer will be low, reducing the expression of proteins driven by this complex. These proteins mediate significant antiviral activity^23^ as well as resistance to DNA damage in cancer cells.^24,25^ Our study of the U-STAT1-U-STAT2 dimer, including structural insights together with demonstration of biochemical and biological significance, provides a greatly expanded understanding of the detailed regulation of IFN-dependent signaling, especially seemingly paradoxical functions of the STAT1-STAT2 dimer as a positive or negative regulator of cytokine responses, depending on the conformation.

In response to IFN-I, the phosphorylation of STAT2 on T404 affects the transcription of most ISGs, rather than a specific subset of these genes. Our ChIP data (Fig. 2c, d) show that the phosphorylation of T404 enhances the affinity of ISGF3 for DNA. Partnering with IRF9, STAT2 contacts the upstream half of ISREs, the “G” in the 5′-GAAA-3′ sub-element, while IRF9 interacts with the following “AAA”.^26^ Although the interaction of ISREs with STAT2 is weaker than their interaction with IRF9, it has been suggested that a key function of STAT2 is to position ISGF3 properly on promoters, stabilizing the following interaction with DNA.^5,27^ This analysis may help to explain why ISGF3 variants containing T404A STAT2 have an altered specificity for ISREs (Fig. 2d), possibly involving conformational changes in ISGF3. The phosphorylation of T404 may alter not only the conformation of the U-STAT1-U-STAT2 dimer, but also the tyrosine phosphorylated STAT1-STAT2 heterodimer within ISGF3.

Patients with STAT2-deficient mutations have an impaired response to IFN-I and suffer from severe viral infections.^28^ Caused by missense mutations of STAT2, patients suffer from the condition called immunodeficiency 44, an autosomal recessive primary immunodeficiency characterized by increased susceptibility to viral infections and encephalopathy.^29,30^ Through ClinVar, we found that one third of the STAT2 mutations occur in the DBD, suggesting that this domain, which includes T404, is critical for a competent immune response, especially against virus infections. A delayed response to IFN-I following virus infection leads to impaired virus clearance, which further transits into an exuberant proinflammatory immune response, called a cytokine storm. Our results with in vivo VSV infections show that a cytokine storm is the main cause of death in mice carrying the T403A mutation of STAT2. This finding emphasizes that this newly discovered phosphorylation of STAT2 is essential for prompt IFN-mediated host defense against virus infections, an effect not limited to VSV.

Clinical reports show that coronavirus 2019-nCoV causes clusters of severe respiratory illness with high mortality.^31^ Several reports demonstrate that multiple coronavirus-encoded structural and non-structural proteins, including ORF3b and ORF6 from severe acute respiratory syndrome coronavirus (SARS-CoV), and ORF4a/b from Middle East respiratory syndrome coronavirus (MERS-CoV), antagonize IFN responses.^32,33^ Additionally, structural proteins such as the membrane (M) proteins, have been shown to inhibit TBK1 and IKK-ε,^34^ which are required for the T404 phosphorylation of STAT2. Most importantly, an early IFN response is protective in SARS-CoV-infected mice.^35^ Therefore, a prompt IFN response is critical in determining the outcome of coronavirus infections. We hope that our findings regarding a novel mechanism of IFN activation that boosts antiviral defenses will aid in designing improved therapeutic approaches to emerging virus infections, including but not limited to coronaviruses.

## Materials and methods

### Reagents

Human IFN-β was from PBL Interferon Source. Recombinant mouse IFN-β1 was from Biolegend. Mouse monoclonal antibodies against STAT2 (Santa Cruz Biotechnology), IRF9 (ISGF3γ) (Santa Cruz Biotechnology), VSV-G (Santa Cruz Biotechnology), Tyr 701-phosphorylated STAT1 (Cell Signaling), and Tyr 690-phophorylated STAT2 (Cell Signaling), STAT1 (Upstate), were used for western blot analyses. Rabbit polyclonal antibodies against IRF9 (Santa Cruz Biotechnology) were used for immunoprecipitations. Staurosporine was from Selleckchem. SureBeads Protein A Magnetic Beads were from Bio-Rad. Anti-FLAG^®^ M2 Magnetic Beads were from Sigma.

### Preparation and purification of antibodies against T404-phosphorylated STAT2 peptides

Anti-pT404 STAT2 antibody was raised by using peptides representing human STAT2 residues 399−411, with and without phosphorylation of T404. The sequence is: DFGYLTLVEQRSG. The antibody was prepared and purified by Shanghai Immune Biotech.

### Mice

STAT2 T403A/T403A mutant mice on a C57BL/6 background were generated by using the CRISPR/Cas9 method. Briefly, the mouse STAT2 gene, located on chromosome 10, was broken and repaired by homologous recombination, using a donor DNA in which a 120 bp homologous arm flanked both sides of the mutant site. After recombination, the ACT sequence encoding a threonine (T) in WT STAT2 was replaced by GCG, encoding an alanine residue. Mice were kept and bred in pathogen-free conditions.

Genotyping of STAT2 T403A/T403A Knock-in Mice. Mouse Stat2(T403A)-F: 5′-GCTTAATTTGGGACTTCGGCTTCT-3′; Mouse Stat2(T403A)-R: 5′-GACACCCACTCCAGGACGCTTC-3′; Product Size: 628 bp; Annealing Temp: 59 °C; DNA Sequencing Primer (Reverse Sequencing): 5′-CCCTTCCTCTAAATCTGCTGCCTCT-3′.

### Culture and maintenance of cells

HEK293T and HeLa cells were obtained from the ATCC and grown in DMEM (Gibco-BRL), supplemented with 5% fetal bovine serum (FBS) with penicillin (100 U/mL), and streptomycin (100 μg/mL). The human normal mammary epithelial cell line hTERT-HME1 was purchased from Clontech and grown in mammary epithelium growth media (MEGM) containing bovine pituitary extract, hydrocortisone, insulin, epithelial growth factor, and gentamycin/amphotericin-B (Lonza). U6A cells were described previously.^36^ WT and STAT2 T403A/T403A MEFs were cultured in Dulbecco’s modified Eagle’s medium (DMEM) plus 10% fetal bovine serum (FBS). WT and STAT2 T403A/T403A BMDCs were cultured in RPMI-1640 medium plus 10% FBS. Vero cells were cultured in DMEM plus 5% FBS. All the media were supplemented with penicillin (100 IU/mL), and streptomycin (100 mg/mL). Cells were maintained in an incubator at 37 °C with 5% CO_2._

### Electron microscopy and image processing

Negatively stained specimens were prepared following an established protocol. Briefly, 3 µL of purified STAT1-STAT2 (WT or T404A) from the peak fraction of a size-exclusion chromatogram were applied to glow-discharged copper EM grids covered with a thin layer of continuous carbon film, and the grids were stained with 2% (w/v) uranyl formate. The grids were imaged on a Tecnai TF20 electron microscope (FEI) operated at 200 keV at a nominal magnification of 50,000× using a 4k by 4k CMOS camera (F416, TVIPS), corresponding to a calibrated pixel size of 1.89 Å on the specimen level. Image processing was carried out using the workflow in cryosparc v2. Defocus values of images were calculated using Patch CTF. Particle picking was performed using Blob picker. 2D classification of extracted particle images were carried out with 100 classes to run 20 iterations. An initial 3D model was generated with selected good 2D class averages by Ab-initio Reconstruction. Heterogeneous refinement of selected good 2D classes was performed with the initial 3D model and decoys generated from junk particles. Homogenous refinement was carried out with the final homogenous set of particles. The overall resolutions were estimated based on the gold-standard Fourier shell correlation (FSC) = 0.143 criterion. The number of final set of particles is 43,760 yielding an 8.4 Å EM map.

### VSV and HSV infection

VSV was propagated and amplified by infection of Vero cells. After infection for 24 h, the supernatant medium was harvested and centrifuged. Virus was titered by using a TCID50 assay. WT, STAT2 WT/T403A, and STAT2 T403/T403A mice (*n* = 20) were injected (I.V.) with 1 × 10^7^ pfu of VSV, or 8 × 10^5^ pfu of HSV. The viability of the infected mice was monitored for 12 days.

### Isolation of MEFs and BMDCs

MEFs from WT and T403A mutant mice were prepared from day 13.5 embryos and cultured in DMEM supplemented with 10% FBS. BMDCs were isolated from the tibias and femurs of mice with different genotypes, and generated using the Inaba method. In brief, bone marrow cells were cultured in RPMI-1640 supplemented with 10% FBS, GM-CSF (20 ng/mL) and IL-4 (10 ng/mL) for 2 days, then 2/3 of the medium was removed and replaced with fresh medium with 10% FBS, GM-CSF (20 ng/mL), and IL-4 (10 ng/mL) for another 4 days. At day 6, the cells were gently aspirated and cell supernatants were collected by centrifugation. The collected cells were seeded in 35 mm dishes at a density of 1 × 10^6^ cells/mL; mature DCs were treated with IFN-β.

### MS analysis of STAT2 PTMs

STAT2 was purified from 293T cells expressing STAT2-Flag by using anti-FLAG^®^ M2 Magnetic Beads (Sigma). For protein digestions, the bands were cut from the gels, washed, and de-stained in 50% ethanol, 5% acetic acid. The gel pieces were then dehydrated in acetonitrile, dried in a Speed-vac, and digested by adding 5 µL of 10 ng/µL of trypsin or chymotrypsin, in 50 mM ammonium bicarbonate, followed by incubation overnight. The peptides were extracted in two portions of 30 µL each of 50% acetonitrile, 5% formic acid. The combined extracts were evaporated to < 10 µL in a Speed-vac and then re-suspended in 1% acetic acid to make a final volume of ~30 µL. The LC-MS system was a Finnigan LTQ-Obitrap Elite hybrid mass spectrometer system. The HPLC column was a Dionex 15 cm × 75 µm id Acclaim Pepmap C18, 2 µm, 100 Å reversed-phase capillary chromatography column. 5 µL volumes of extract was injected and the peptides, eluted from the column in an acetonitrile, 0.1% formic acid gradient at a flow rate of 0.25 µL/min, were introduced into the source of the MS online. The micro-electrospray ion source was operated at 2.5 kV. The digest was analyzed in both survey and targeted manners. Survey experiments were performed using the data-dependent multitask capability of the instrument, acquiring full-scan mass spectra to determine peptide molecular weights and product ion spectra to determine amino acid sequences in successive instrument scans. The LC-MS/MS data was searched with the Mascot and Sequest programs against both the full human reference sequence database and specifically against the sequence of STAT2. The parameters used include a peptide mass accuracy of 10 ppm, fragment ion mass accuracy of 0.6 Da, carbamidomethylated cysteines as a constant modification, and oxidized methionine and phosphorylation at S, T, and Y as a dynamic modification. The results were filtered based on Mascot ion scores and Sequest XCorr scores. All positively identified phosphopeptides were validated manually. The targeted experiments involve the analysis of specific STAT2 peptides, including the phosphorylated and unmodified forms of the T404 peptides. The chromatograms for these peptides were plotted based on known fragmentation patterns and the peak areas of these chromatograms were used to determine the extent of phosphorylation.

### RNA interference

Constructs in the lentiviral vector pLV-tetO-CMV-SV40-Puro-LoxP capable of expressing human STAT2 or IRF9 were described previously.^23^ shRNAs in the lentiviral vector pLKO against IKK-ε and TBK1 were obtained from Sigma-Aldrich. shTBK1-1, TRCN0000314787; shTBK1-2, TRCN0000314839; shIKBKE-1, TRCN0000010035; shIKBKE-2, TRCN0000010037.

### Cell growth assay

Cell cultures were assayed for survival using the Sigma MTT method, according to the manufacturer’s instructions. Briefly, the reagent was added directly to the culture medium. After 4 h, the medium was removed, neat DMSO was added to extract the reagent, and the signal was read with a fluorometer.

### RNA isolation and qRT-PCR assay

Total RNA was extracted from cells using the TRIzol reagent (Invitrogen) according to the manufacturer’s protocol. cDNA was synthesized from total RNA using a modified manufacturer’s protocol, with random hexamer and Superscript III (Invitrogen). Real-time PCR was performed with EvaGreen qPCR master mix (Bullseye) in a LightCycler 480 (Roche). The PCR protocol was: initial activation at 95 °C for 5 min, 40 cycles at 95 °C for 15 s and 60 °C for 1 min. Ct values were converted into relative gene expression levels, compared to that of the internal control genes GAPDH or 18 s RNA, using the ΔΔCt method or the standard curve method.^37^

The specificity was confirmed by analysis of the melting curves of the PCR products. The primer sequences used were as follows: OAS1-F, TGAGGTCCAGGCTCCACGCT; OAS1-R, GCAGGTCGGTGCACTCCTCG; IFIT1-F, TCTCAGAGGAGCCTGGCTAA; IFIT1-R, CCAGACTATCCTTGACCTGATGA; IFIT3-F, CAGAACTGCAGGGAAACAGC; IFIT3-R, TGAATAAGTTCCAGGTGAAATGGC; DDX58-F, AAACAAATCAGAACACAGGAATG; DDX58-R, CCTCTGCCTCTGGTTTGG; m-IFIT1-F, CAGAAGCACACATTGAAGAA; m-IFIT1-R, TGTAAGTAGCCAGAGGAAGG; m-IFIT2-F, CGGAAAGCAGAGGAAATCAA; m-IFIT2-R, TGAAAGTTGCCATACCGAAG; m-Mx1-F, TAATCTGTGCAGGCACTATGAGG; m-Mx1-R, AGAGAGCTCCACTTCAATGTCATC; VSV N/P-F, CAAAACAGAAAACCGACTCC; VSV N/P-R, AGACGAGAATAGGACTTGAGA; m-18S rRNA-F, ATTGACGGAAGGGCACCACCAG; m-18S rRNA-R, CAAATCGCTCCACCAACTAAGAACG; m-IFN-b-F, CTTCTCCGTCATCTCCATAGGG; m-IFN-b-R, CACAGCCCTCTCCATCAACT

### Viral infection and TCID50 assay

VSV and VSV-GFP viruses were titrated as previously described.^38^ For infections, cells were cultured in serum-free medium, and the viruses were inoculated at the multiplicities of infection indicated in the figures. Two hours later, cells were washed with PBS and fresh culture medium was added. Cells were harvested 20 h after infection. The TCID50 assay protocol was followed as described in the chapter “Principles of Virology” from the textbook Fields Virology.

### Tissue digestion and flow analysis

Four days post infection, mice were sacrificed and the brains were digested with the mouse Adult Brain Dissociation Kit (Miltenyi Biotec) according to the manufacturer’s protocol. Briefly, half of the brain was cut into ~4 sagittal slices and transferred into C Tubes containing 2 mL of enzyme mix. Tissue disruption was done in a C Tube, using a gentleMACS Dissociator (Miltenyi Biotec) using program 37C_ABDK_01. After digestion, enzymes were removed by washing with PBS and the digested tissues were pelleted by centrifugation. Cell pellets were carefully resuspended with 3.1 mL cold D-PBS for removal of debris and red blood cells. The remaining single cell suspensions were treated with blocking buffer (20% FBS, 1:100 CD16/CD32 antibodies and 1:100 Rat IgG) for 20 min and then stained with antibodies for 30 min at 4 °C. The following antibodies were used for staining: CD45-PerCP-Cy5.5, CD11b-PE, CD206 PE-Cy7, Ly6C-APC, CD4-BV510, CD19-APC, CD8-PerCP-Cy5.5 and CD69-PE. The samples were washed, and signals were acquired by FACS Arial III (BD Biosciences) and analyzed by FlowJo 10.4.

### Immunoprecipitations

Cell pellets were suspended in a nuclear extraction buffer containing 50 mM Tris·HCl, pH 7.4, 150 mM NaCl, 1 mM EDTA, 0.5% TRITON^®^ X-100 with proteinase/phosphatase inhibitors. Whole cell lysates (1 mg) were precleared with Protein A/G PLUS Agarose (Santa Cruz) and incubated with 3 μg of mouse monoclonal antibodies overnight. The antibody-bound proteins were precipitated with SureBeads Protein A Magnetic Beads (Bio-Rad), washed with PBS, and boiled with loading buffer containing 0.9% β-mercaptoethanol.

### Western blot assay

Whole-cell extracts were prepared by incubating cell pellets in lysis buffer containing 50 mM Tris·HCl, pH 7.4, 150 mM NaCl, 1 mM EDTA, 0.5% TRITON® X-100, and a mixture of protease and phosphatase inhibitors (Roche). After incubation on ice for 20 min, cell debris was removed by centrifugation. Cell extracts containing equal quantities of proteins, determined by the Bradford method, were separated by SDS/PAGE (10% (v/v) acrylamide) and transferred to polyvinylidene difluoride membranes (Millipore). The membranes were incubated with primary antibody for 2 h, followed by incubation with secondary antibody for 1 h at room temperature, and developed by using the enhanced chemiluminescence solution (Perkin-Elmer).

### Gene expression analysis

Total RNA was isolated by using a Qiagen RNeasy Mini Kit according to the manufacturer’s instructions, and 1 μg of this RNA was used for microarray analysis on an Illumina HumanHT-12 v4 Expression BeadChip Kit. Data were analyzed by using the Illumina BeadStudio software and normalized by the quantile method. Genes were selected that satisfied the following criteria: differential *P* values of ≤ 0.01, average signals > 25, and signals that changed by > 2-fold. The average signal for each probe was used to determine expression levels. Genes with average signals below 25 and detection *P* values greater than 0.01 in the untreated or treated cells were excluded from the analysis. Inductions of less than 2-fold were not scored. The microarray data from this publication have been submitted to the GEO database and assigned the GEO accession number GSE126565.

### ChIP analysis

These assays were performed with the SimpleChIP® Enzymatic Chromatin IP Kit (Cell Signaling), according to the manufacturer’s instructions. The cells were lysed, and the chromatin was fragmented to 320-bp pieces by digestion with Micrococcal Nuclease. DNA/protein complexes were precipitated by overnight incubation with 4 µg of anti-IRF9, anti-histone H3, or normal rabbit anti-IgG, and then incubated with Protein A/G agarose beads for 2 h. After reversal of protein-DNA cross-links, the DNA was purified and the abundance of the *ADAR1* and *MX1* promoters was analyzed by qPCR. The sequences of primers spanning ISREs on the gene promoter are shown below. The ChIP-seq data from this publication have been submitted to the BioProject database and assigned the accession number PRJNA522441. ChIP-MX1-F: GGGACAGGCATCAACAAAGCC; ChIP-MX1-R: GCCCTCTCTTCTTCCAGGCAAC; ChIP-ADAR-F: AGCGGAGTGGTAAGACCAGA; ChIP-ADAR-R: GCCTGAGCTGAGACTGCAA.

### EMSA

Nuclear extracts were prepared with the Nuclear Extract Kit (Active Motif). The probe was synthesized and labeled with the biotin on 3′ end. ISRE (ISG15), 5′‐GGGAAAGGGAAACCGAAACTGA‐3′ and 5′‐TCAGTTTCGGTTTCCCTTTCCC‐3′. The DNA binding reaction and native polyacrylamide gel electrophoresis were performed following the instructions for Gelshift Chemiluminescent EMSA (Active Motif). The competition EMSA was done as described, using a 500-fold molar excess of unlabeled probe.

### RNA-seq analysis

Total RNA was isolated and used for RNA-seq analysis. cDNA library construction and sequencing were performed by Qingdao OE Biotechnology Co., Ltd using the Illumina Hiseq Xten platform. Clean reads were aligned to the mouse reference genome (GRCm38) using hisat2. The expression levels for each of the genes were normalized to fragments per kb of exon model per million mapped reads (FPKM) using RNA-seq by Expectation Maximization (RSEM). The RNA-seq data from this publication have been submitted to the BioProject database and assigned the accession number PRJNA609324.

### In vitro kinase assay

The kinase buffer contains 50 mM HEPES, 0.01% Tween 20, 10 mM MnCl_2_, 1 mM EGTA, 2.5 mM DTT and 0.1 mM ATP, pH 7.4. Myc-tagged kinases and Flag-tagged STAT2 were purified from 293T cells. Kinases and substrates were added to the reaction buffer for 60 min at 30 °C. All reactions were stopped by adding 5× SDS loading buffer and boiled for 10 min at 95 °C for western blot analysis.

### Quantification and statistical analysis

Results from ChIP and luciferase assays are represented by means ± SEM. Data were analyzed by two-way ANOVA. *p* values of ≤ 0.05 are considered statistically significant. *P ≤ 0.05, **P ≤ 0.01, ***P ≤ 0.001, non-significant (ns), *P* > 0.05.

## Supplementary information

Supplementary information, Fig. S1

Supplementary information, Fig. S2

Supplementary information, Fig. S3

Supplementary information, Fig. S4

Supplementary information, Fig. S5

Supplementary information, Table S1
