# Supplementary material for: A virus-induced conformational switch of STAT1-STAT2 dimers boosts antiviral defenses
Source: Cell Res. 2020 Aug 5;31(2):206–18. doi: 10.1038/s41422-020-0386-6 (PMC7405385; doi:10.1038/s41422-020-0386-6)
Supplement: Supplementary file 1 — Supplementary information, Fig. S1 [file 41422_2020_386_MOESM1_ESM.pdf]

Fig S1. Conformational analysis of U-STAT1-U-STAT2 dimer.

a. (i) Size-exclusion chromatography profile for STAT1-STAT1 homodimer and STAT1-STAT2 heterodimer. The fraction used for EM analysis is highlighted in red rectangle box. (ii) Two-dimension class averages of particles from negative stain EM micrographs. (iii) Fourier shell correlation (FSC) curve of the final EM map and the angular distribution of particles.
b. Western analysis of STAT2 from a GST pull-down assay, using HEK293T cells transfected with GST-tagged truncations of STAT1.
c. Western analysis of STAT1 from a GST pull-down assay, using HEK293T cells transfected with truncations of GST-tagged STAT2.
d. EM density of STAT1-STAT2 dimers contain WT-STAT2 (contour level of 1.81) shown in blue, and T404A-STAT2 (contour level of 1.17) shown in orange. Correlation=0.9835, Overlap = 1.85e+05.
e. U3A cells expressing WT, F77A, F172A, Q340W, or Y701F STAT1 were treated with IFN-β (100 IU/ml) for 30 min or were untreated. Whole-cell lysates were analyzed by the Western method.

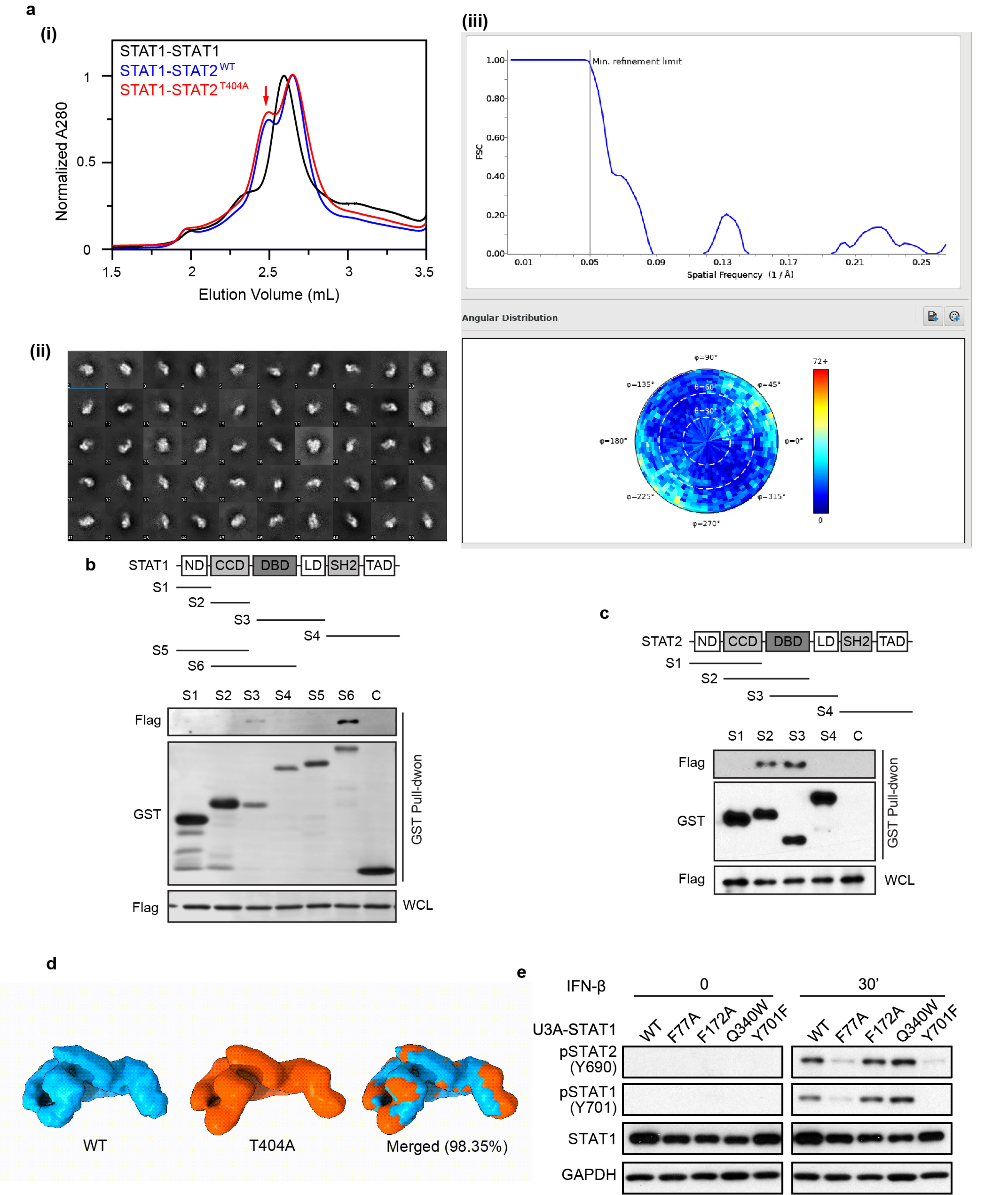
a
(i)
STAT1-STAT1
STAT1-STAT2 WT
STAT1-STAT2 T404A
Normalized A280
Elution Volume (mL)
1
0.5
0
1.5
2
2.5
3
3.5
(ii)
(iii)
Min. refinement limit
FSC
Spatial Frequency (1 / Å)
1.00
0.80
0.60
0.40
0.20
0.00
0.01
0.05
0.09
0.13
0.17
0.21
0.25
Angular Distribution
φ=90°
φ=135°
φ=45°
θ=60°
θ=30°
φ=180°
φ=0°
φ=225°
φ=315°
φ=270°
72+
0
b
STAT1
ND
CCD
DBD
LD
SH2
TAD
S1
S2
S3
S4
S5
S6
C
Flag
GST
GST Pull-dwon
WCL
c
STAT2
ND
CCD
DBD
LD
SH2
TAD
S1
S2
S3
S4
C
Flag
GST
GST Pull-dwon
WCL
d
WT
T404A
Merged (98.35%)
e
IFN-β
0
30'
U3A-STAT1
WT
F77A
F172A
Q340W
Y701F
pSTAT2 (Y690)
pSTAT1 (Y701)
STAT1
GAPDH
