# Supplementary material for: A virus-induced conformational switch of STAT1-STAT2 dimers boosts antiviral defenses
Source: Cell Res. 2020 Aug 5;31(2):206–18. doi: 10.1038/s41422-020-0386-6 (PMC7405385; doi:10.1038/s41422-020-0386-6)
Supplement: Supplementary file 2 — Supplementary information, Fig. S2 [file 41422_2020_386_MOESM2_ESM.pdf]

Fig S2. Phosphorylation of STAT2 on T404 expedites the tyrosine phosphorylation of STAT1 and STAT2, and enhances the DNA-binding activity of ISGF3.

a. STAT2-null U6A cells expressing WT or T404A STAT2 were treated with IFN-β (100 IU/ml) for 0, 4, 8, or 24 h. Total RNAs were analyzed by qRT-PCR.
b. U6A cells expressing WT, T404A, T404D, or T404E STAT2 were treated with IFN-β (100 IU/ml). Cells were harvested after 4 h and total RNAs were analyzed by qRT-PCR.
c. Venn diagram of IFN-induced genes from (Fig. 2b) that were affected by 0.8-fold or less in U6A cells expressing T404A STAT2 than in cells expressing WT STAT2.
d. Scatterplot diagram of genes down regulated in U6A-T404A-STAT2 cells relative to U6A-WT-STAT2 cells following treatment with IFN-β. Red lines represent 2-fold changes.
e. EMSAs using an ISRE probe. Nuclear extracts from U6A cells expressing empty vector (V), WT (W), T404A (A) or T404E (E) STAT2 treated with IFN-β for 1 h were analyzed. Neg: Nuclear extracts (WT-STAT2 treated with IFN-β) with unlabeled competitive probe. S1, S2, I9: Nuclear extracts (WT-STAT2 treated with IFN-β) with STAT1, STAT2 or IRF9 antibody.
f. U6A cells expressing WT, T404A or T404E STAT2 were treated with IFN-β (100 IU/ml) for 0 or 30 min, or 24 h. Whole-cell lysates were analyzed by the Western method.
g. HME cells expressing empty vector (V), WT (W), T404A (A) or T404E (E) STAT2 were treated with IFN-β (100 IU/ml) for 0, 4, or 9 h. Whole-cell lysates were harvested and analyzed by the Western method.
h. U6A cells expressing WT, T404A, or T404E STAT2 were treated with IFN-β (100 IU/ml) for 2 h or were untreated. The cells were washed with PBS and the medium was replaced with fresh medium containing Staurosporine (500 nM). Whole-cell lysates were harvested and analyzed by the Western method.
i. U6A cells expressing WT, T404A, or T404E STAT2 were treated with IFN-β (100 IU/ml) for 30 min or 48 h, or were untreated. Cytoplasmic and nuclear extracts were analyzed by the Western method.

Data are shown as means ± SEM from three independent experiments. P-values were calculated using the paired ratio t-test (**P* < 0.05, ***P* < 0.01, ****P* < 0.001, NS, not significant).

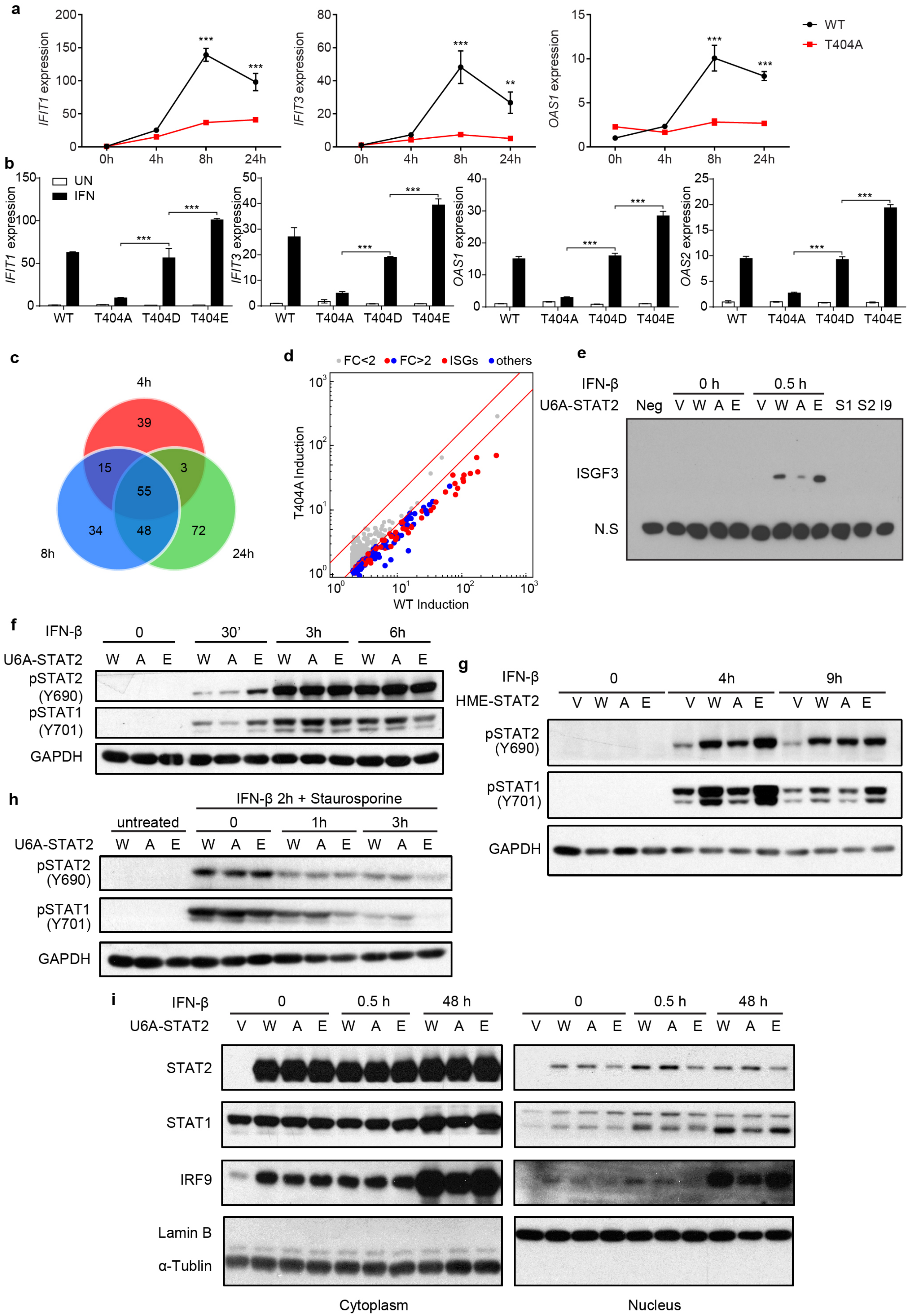
a
IFIT1 expression
IFIT3 expression
OAS1 expression
0h
4h
8h
24h
WT
T404A
b
UN
IFN
OAS2 expression
WT
T404A
T404D
T404E
c
4h
8h
24h
39
15
3
55
34
48
72
d
FC<2
FC>2
ISGs
others
T404A Induction
WT Induction
e
IFN-β
0 h
0.5 h
U6A-STAT2
Neg
V W A E
V W A E
S1 S2 I9
ISGF3
N.S
f
IFN-β
0
30'
3h
6h
U6A-STAT2
pSTAT2 (Y690)
pSTAT1 (Y701)
GAPDH
g
IFN-β
0
4h
9h
HME-STAT2
h
IFN-β 2h + Staurosporine
untreated
0
1h
3h
i
IFN-β
0
0.5 h
48 h
STAT2
STAT1
IRF9
Lamin B
α-Tublin
Cytoplasm
Nucleus
