# Supplementary material for: A virus-induced conformational switch of STAT1-STAT2 dimers boosts antiviral defenses
Source: Cell Res. 2020 Aug 5;31(2):206–18. doi: 10.1038/s41422-020-0386-6 (PMC7405385; doi:10.1038/s41422-020-0386-6)

Fig S3. IKK-ε is a kinase for STAT2 T404 phosphorylation.

a. ELISA analysis of an antibody that recognizes phosphorylated human STAT2 T404.
b. Purified Flag-STAT2 was mixed with the commercial TBK1, immunoprecipitated with anti-Flag, then immunoblotted with anti-TBK1
c. Western analysis of STAT2 from a GST pull-down assay, using HEK293T cells transfected with truncations of GST-tagged IKK-ε. KD: Kinase Domain; ULD: Ubiquitin-like Domain; LZ: Leucine Zipper; HLH: Helix-Loop-Helix.
d. Western analysis of IKK-ε from a GST pull-down assay, using HEK293T cells co-transfected with truncations of GST-tagged STAT2 and myc-tagged IKK-ε. ND: N-terminal Domain; CCD: Coiled-coil Domain; DBD: DNA Binding Domain; LD: Linker Domain; SH2: Src-Homology 2 Domain; TAD: Trans-Activation Domain.
e. Whole-cell lysates from Hela cells expressing shRNAs targeting IKK-ε were analyzed by the Western method.
f. Hela cells expressing shRNAs targeting IKK-ε were treated with IFN-β (100 IU/ml) for 4 h. Total RNA was analyzed by qRT-PCR.
g. Whole-cell lysates from Hela cells expressing shRNAs targeting TBK1 were analyzed by the Western method.
h. Hela cells expressing shRNAs targeting TBK1 were treated with IFN-β (100 IU/ml) for 4 h. Total RNA was analyzed by qRT-PCR.
i. HME cells were infected with VSV (MOI=1) or HSV (MOI=5) for 2 or 6 h. Whole-cell lysates were analyzed by the Western method.

Data are shown as means ± SEM from three independent experiments. P-values were calculated using the paired ratio t-test ($*P < 0.05$, $**P < 0.01$, $***P < 0.001$, NS, not significant).

a

Antigen for ELISA	Blank	1:3.125K	1:6.25K	1:12.5K	1:25K	1:50K	1:100K
DFGYL(pT)LVEQRSG-C	0.056	2.499	2.133	1.639	1.027	0.664	0.377
DFGYLTLVEQRSG-C	0.067	0.113	0.094	0.099	0.082	0.067	0.055

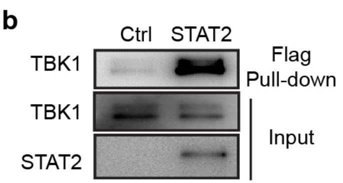

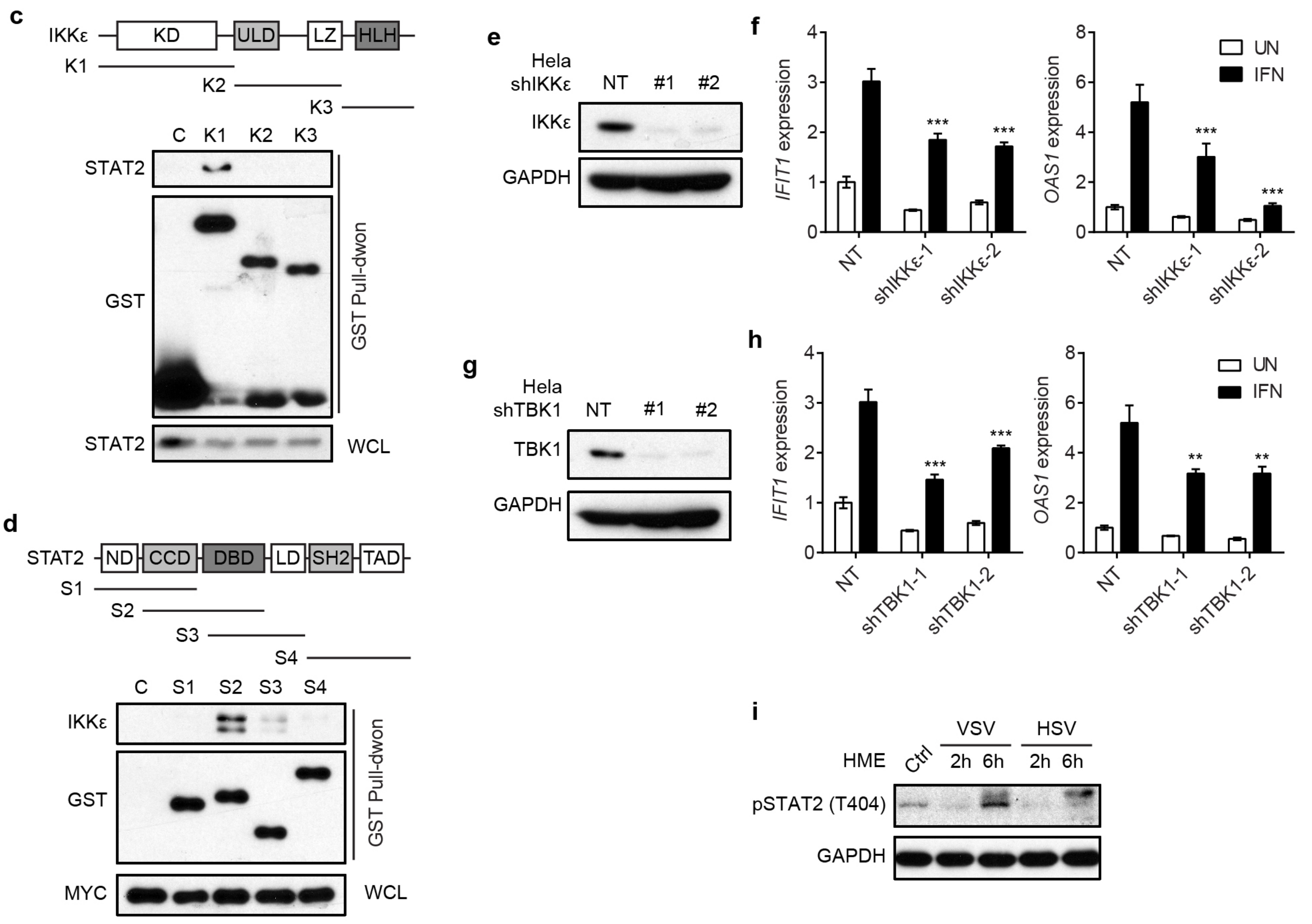

Supplement: Supplementary file 3 — Supplementary information, Fig. S3 [file 41422_2020_386_MOESM3_ESM.pdf]
