# Supplementary material for: A virus-induced conformational switch of STAT1-STAT2 dimers boosts antiviral defenses
Source: Cell Res. 2020 Aug 5;31(2):206–18. doi: 10.1038/s41422-020-0386-6 (PMC7405385; doi:10.1038/s41422-020-0386-6)
Supplement: Supplementary file 4 — Supplementary information, Fig. S4 [file 41422_2020_386_MOESM4_ESM.pdf]

Fig S4. Insufficient IFN-I response in T403A/T403A mice.

a. Schematic diagram showing the conservation of sequences surrounding STAT2 T404 in mammals.
b. To generate the T403A mutation, the mouse STAT2 gene, located on chromosome 10, was broken by using CRISPR/CAS9 and repaired by homologous recombination, using a donor DNA in which a 120 bp homologous arm flanked both sides of the mutant site. After recombination, the ACT codon, encoding threonine (T) in wild type STAT2, was replaced by a GCG codon, encoding Alanine (A).
c. DNA sequences around the mutant site in (a) were amplified by PCR and identified by DNA sequencing in WT and STAT2 T403A mice, respectively.
d. Primary MEFS with WT or T403A STAT2 were treated with mouse IFN-β (100 IU/ml) for 0, 4, 8, or 12 h, and total RNA was analyzed by qRT-PCR.
e. Primary BMDCs with WT or T403A STAT2 were treated with mouse IFN-β (100 IU/ml) for 0, 4, 8, or 12 h, and total RNA was analyzed by qRT-PCR.
f. Gene ontology analysis of differentially expressed genes in Fig 4a, right.

Data are shown as means ± SEM from three independent experiments. P-values were calculated using the paired ratio t-test (**P* < 0.05, ***P* < 0.01, ****P* < 0.001, NS, not significant).

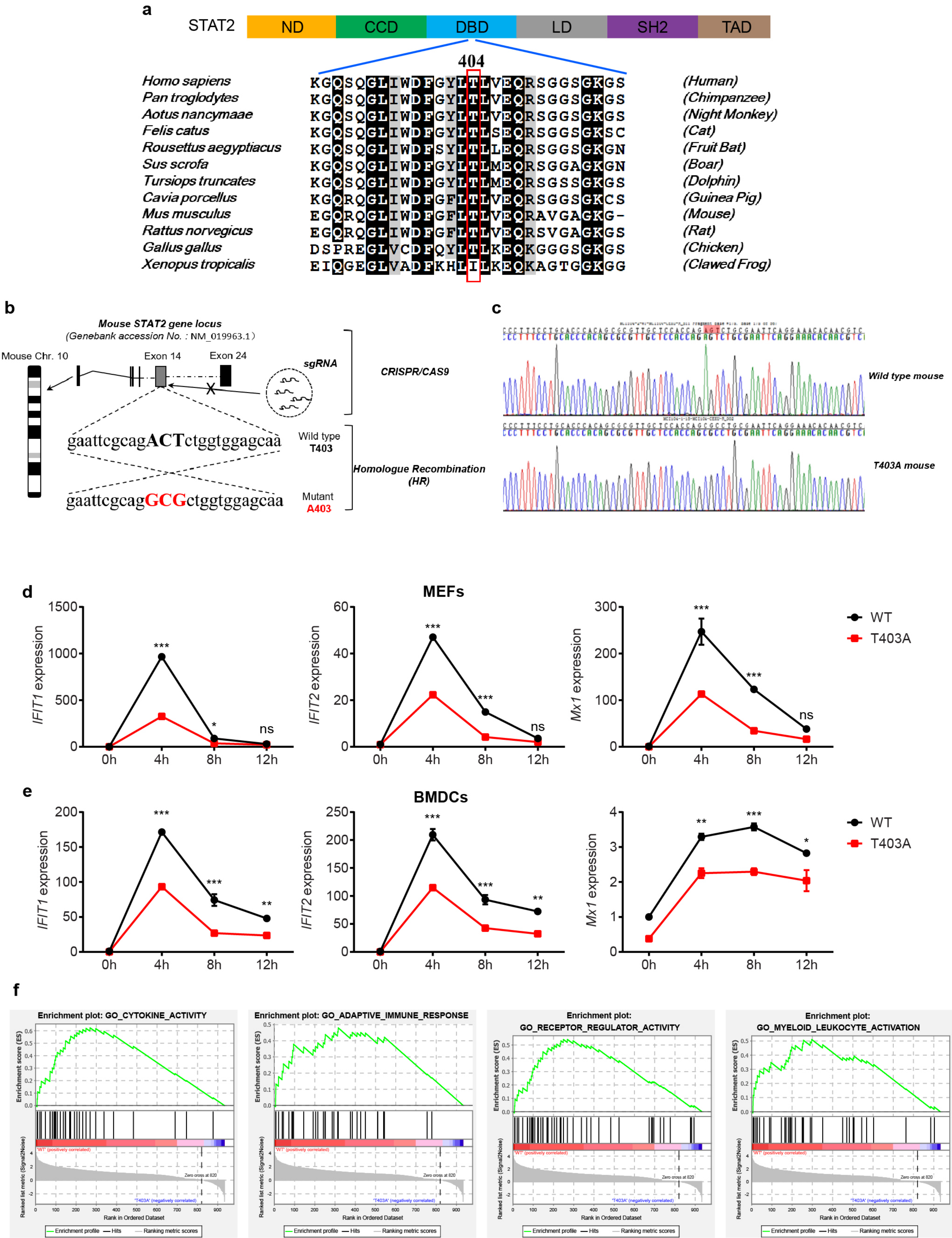
a
STAT2
ND
CCD
DBD
LD
SH2
TAD
404
Homo sapiens KGQSQGLIWDFGYLTLVEQRSGGSGKGS (Human)
Pan troglodytes KGQSQGLIWDFGYLTLVEQRSGGSGKGS (Chimpanzee)
Aotus nancymaae KGQSQGLIWDFGYLTLVEQRSGGSGKGS (Night Monkey)
Felis catus KGQSQGLIWDFGYLTLSEQRSGGSGKSC (Cat)
Rousettus aegyptiacus KGQSQGLIWDFSYLTLLEQRSGGSGKGN (Fruit Bat)
Sus scrofa KGQSQGLIWDFGYLTLMEQRSGGAGKGN (Boar)
Tursiops truncates KGQSQGLIWDFGYLTLMEQRSGSSGKGS (Dolphin)
Cavia porcellus KGQRQGLIWDFGFLTLVEQRSGGSGKCS (Guinea Pig)
Mus musculus EGQRQGLIWDFGFLTLVEQRAVGAGKG- (Mouse)
Rattus norvegicus EGQRQGLIWDFGFLTLVEQRSVGAGKGS (Rat)
Gallus gallus DSPREGLVCDFQYLTLKEQKGGGSGKGS (Chicken)
Xenopus tropicalis EIQGEGLVADFKHLILKEQKAGTGGKGG (Clawed Frog)
b
Mouse STAT2 gene locus
(Genebank accession No. : NM_019963.1)
Mouse Chr. 10
Exon 14
Exon 24
sgRNA
CRISPR/CAS9
gaattcgcagACTctggtggagcaa
Wild type
T403
Homologue Recombination
(HR)
gaattcgcagGCGctggtggagcaa
Mutant
A403
c
Wild type mouse
T403A mouse
d
MEFs
IFIT1 expression
IFIT2 expression
Mx1 expression
0h 4h 8h 12h
WT
T403A
e
BMDCs
IFIT1 expression
IFIT2 expression
Mx1 expression
0h 4h 8h 12h
WT
T403A
f
Enrichment plot: GO_CYTOKINE_ACTIVITY
Enrichment plot: GO_ADAPTIVE_IMMUNE_RESPONSE
Enrichment plot: GO_RECEPTOR_REGULATOR_ACTIVITY
Enrichment plot: GO_MYELOID_LEUKOCYTE_ACTIVATION
Enrichment score (ES)
Ranked list metric (Signal2Noise)
Rank in Ordered Dataset
Enrichment profile
Hits
Ranking metric scores
