# Supplementary material for: A virus-induced conformational switch of STAT1-STAT2 dimers boosts antiviral defenses
Source: Cell Res. 2020 Aug 5;31(2):206–18. doi: 10.1038/s41422-020-0386-6 (PMC7405385; doi:10.1038/s41422-020-0386-6)
Supplement: Supplementary file 5 — Supplementary information, Fig. S5 [file 41422_2020_386_MOESM5_ESM.pdf]

Fig S5. T403A/T403A mice are highly susceptible to virus infection.

a. Primary MEFs were pre-treated with 10 IU/ml IFN-β for 2 h, then infected with VSV-GFP (MOI=1) for 2 h. Cells were monitored with a fluorescence microscope at 12 h post infection. Scale bar=50μm.
b. HME cells expressing wild-type, T404A, or T404E STAT2 were seeded at 8000 cells/well. The cells were exposed to VSV-GFP for 2 h, with or without pre-treatment with IFN-β (100 IU/ml). After 20 h, the expression of GFP was analyzed by fluorescence microscopy. Scale bar=50μm.
c. Photos of WT/WT and T403A/T403A mice after VSV infection.
d. WT/WT and T403A/T403A mice (n=4) were infected with VSV for 8 h and the induction of *IFNB* in different tissues was analyzed by qRT-PCR.
e. WT/WT, T403A/WT and T403A/T403A mice (n=4) were infected with VSV for 5 days, the VSV genomic RNA in different organs was measured by qRT-PCR.
f. Differential pathology of WT/WT and T403A/T403A mice in response to VSV. Hematoxylin and eosin staining of different tissues from the mice in (e). Scale bar=100μm.
g. WT or STAT2 T403A mice were injected intravenously with 1 x 10^7 PFU of VSV or buffer. Four days post-infection (dpi), the brains were excised and the cells were analyzed by flow cytometry.
h. Plasma from experiment (g) were analyzed by ELISA.

Data are shown as means ± SEM from two independent experiments. P-values were calculated using the paired ratio t-test (*$P < 0.05$, **$P < 0.01$, ***$P < 0.001$, NS, not significant).

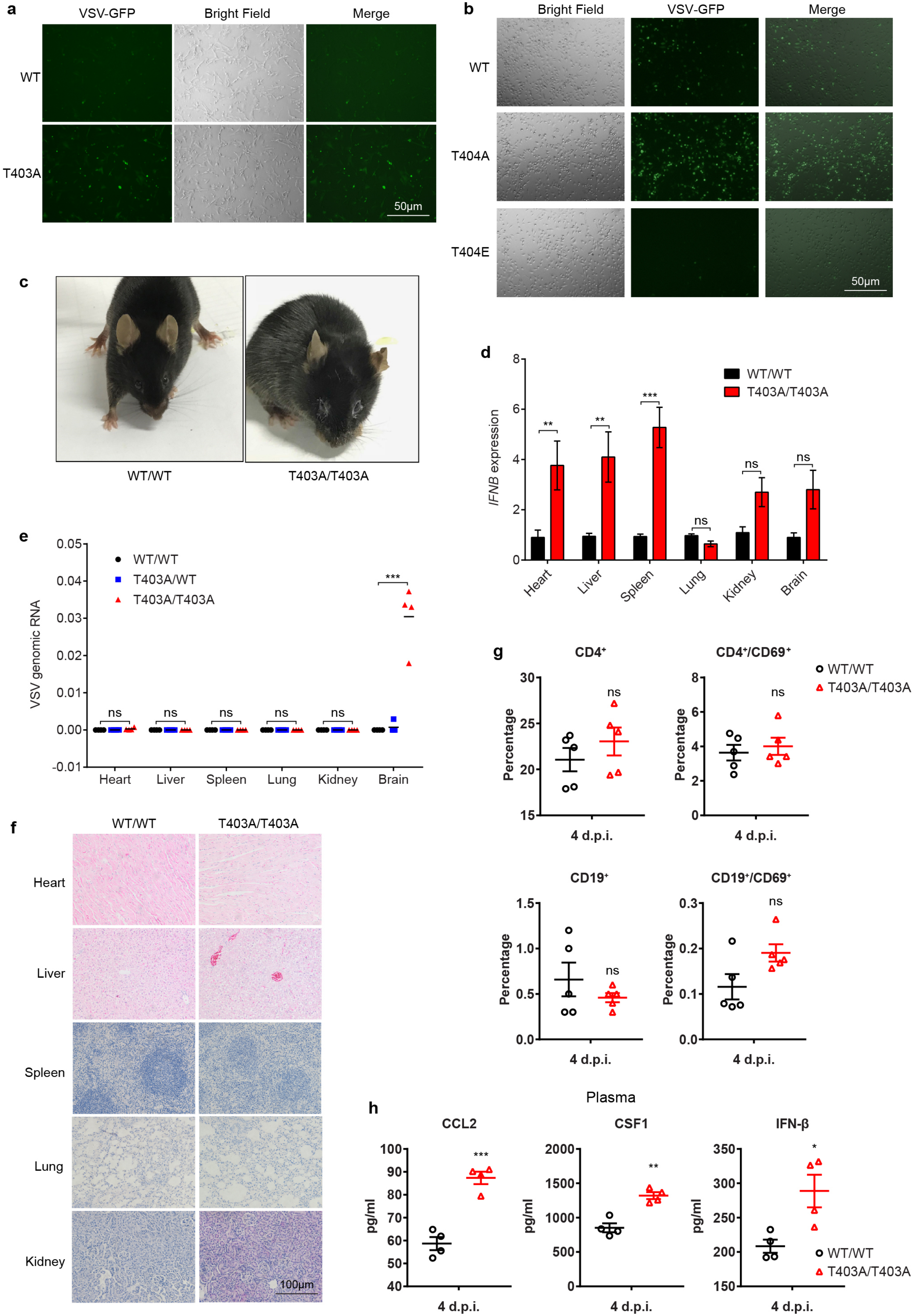

a
VSV-GFP
Bright Field
Merge
WT
T403A
50μm
b
Bright Field
VSV-GFP
Merge
WT
T404A
T404E
50μm
c
WT/WT
T403A/T403A
d
IFNB expression
WT/WT
T403A/T403A
**
**
***
ns
ns
ns
Heart
Liver
Spleen
Lung
Kidney
Brain
e
VSV genomic RNA
WT/WT
T403A/WT
T403A/T403A
***
ns
Heart
Liver
Spleen
Lung
Kidney
Brain
f
WT/WT
T403A/T403A
Heart
Liver
Spleen
Lung
Kidney
100μm
g
CD4+
CD4+/CD69+
CD19+
CD19+/CD69+
WT/WT
T403A/T403A
Percentage
ns
4 d.p.i.
h
Plasma
CCL2
CSF1
IFN-β
pg/ml
***
**
*
WT/WT
T403A/T403A
4 d.p.i.
