# Supplementary material for: A virus-induced conformational switch of STAT1-STAT2 dimers boosts antiviral defenses
Source: Cell Res. 2020 Aug 5;31(2):206–18. doi: 10.1038/s41422-020-0386-6 (PMC7405385; doi:10.1038/s41422-020-0386-6)
Supplement: Supplementary file 6 — Supplementary information, Table S1 [file 41422_2020_386_MOESM6_ESM.pdf]

Table S1. MicroArray Heatmap.

STAT2-null U6A cells expressing WT or T404A STAT2 were treated with IFN-β for 0, 4, 8, or 24 h. Total RNAs were analyzed by using an Illumina HumanHT-12 v4 Expression BeadChip array. The average signal for each probe was used to determine expression levels. Genes with detection *P* values greater than 0.01 in the untreated or treated cells were excluded from the analysis. Inductions of less than 2-fold were not scored. The Venn diagram shows the numbers of genes induced by 0.8-fold or less in U6A cells expressing T404A STAT2, relative to untreated control cells. The numbers in the table are fold changes, calculated from the ratios T404A STAT2 treated / untreated and WT STAT2 treated / untreated.

	4h	8h	24h
ADAR	0.68	0.65	0.85
AIM2	0.25	0.16	0.18
APOL3	0.80	0.54	0.46
BATF2	0.29	0.35	0.39
C19orf66	0.61	0.56	0.79
CASP7	0.70	0.63	0.60
CXCL10	0.76	0.17	0.33
DDX58	0.37	0.31	0.32
DTX3L	0.59	0.43	0.57
EIF2AK2	0.67	0.60	0.74
FAM46A	0.58	0.50	0.62
FST	0.63	0.49	0.51
GBP1	0.66	0.40	0.44
HES4	0.70	0.49	0.50
IFI27	0.56	0.46	0.55
IFI35	0.47	0.41	0.39
IFI44	0.34	0.40	0.47
IFI6	0.30	0.31	0.39
IFIH1	0.31	0.32	0.47
IFIT1	0.37	0.50	0.50
IFIT2	0.16	0.23	0.33
IFIT3	0.28	0.28	0.32
IFIT5	0.53	0.36	0.53
IFITM1	0.30	0.31	0.34
IRF9	0.60	0.55	0.47
ISG15	0.36	0.41	0.40
MASTL	0.76	0.52	0.51

	4h	8h	24h
MX1	0.33	0.34	0.40
MX2	0.25	0.27	0.18
OAS1	0.33	0.33	0.33
OAS2	0.62	0.35	0.29
OAS3	0.64	0.59	0.67
OASL	0.30	0.26	0.28
PARP10	0.67	0.51	0.51
PARP14	0.36	0.36	0.36
PARP9	0.43	0.50	0.70
PLSCR1	0.46	0.43	0.31
PRIC285	0.53	0.46	0.50
PRKD2	0.75	0.61	0.74
REC8	0.45	0.69	0.40
RND3	0.43	0.48	0.60
RSAD2	0.34	0.21	0.26
SAMD9	0.30	0.37	0.52
SAMD9L	0.50	0.40	0.37
SP110	0.59	0.62	0.67
STAT1	0.43	0.38	0.36
STAT2	0.59	0.56	0.58
TAP1	0.52	0.38	0.38
TAP2	0.45	0.53	0.64
TMEM140	0.58	0.22	0.41
TRIM21	0.52	0.50	0.51
TRIM38	0.55	0.36	0.71
UBA7	0.41	0.55	0.46
ZC3HAV1	0.76	0.58	0.88
ZNFX1	0.62	0.66	0.55

1.5
1.4
1.3
1.2
1.1
1.0
0.9
0.8
0.7
0.6
0.5
0.4
0.3
0.2
0.1

$$Relative\ Fold\ Change = \frac{Induction\ Fold\ in\ T404A}{Induction\ Fold\ in\ WT}$$
